# Distinct hyperactive RAS/MAPK alleles converge on common GABAergic interneuron core programs

**DOI:** 10.1242/dev.201371

**Published:** 2023-05-31

**Authors:** Sara J. Knowles, April M. Stafford, Tariq Zaman, Kartik Angara, Michael R. Williams, Jason M. Newbern, Daniel Vogt

**Affiliations:** ^1^ School of Life Sciences, Arizona State University, Tempe, AZ 85287, USA; ^2^ Department of Pediatrics and Human Development, Michigan State University, Grand Rapids, MI 49503, USA; ^3^ Neuroscience Program, Michigan State University, East Lansing, MI 48825, USA

**Keywords:** MAPK, Cortical interneuron, Somatostatin, RASopathy, *bRaf*, *Nf1*

## Abstract

RAS/MAPK gene dysfunction underlies various cancers and neurocognitive disorders. Although the roles of RAS/MAPK genes have been well studied in cancer, less is known about their function during neurodevelopment. There are many genes that work in concert to regulate RAS/MAPK signaling, suggesting that if common brain phenotypes could be discovered they could have a broad impact on the many other disorders caused by distinct RAS/MAPK genes. We assessed the cellular and molecular consequences of hyperactivating the RAS/MAPK pathway using two distinct genes in a cell type previously implicated in RAS/MAPK-mediated cognitive changes, cortical GABAergic interneurons. We uncovered some GABAergic core programs that are commonly altered in each of the mutants. Notably, hyperactive RAS/MAPK mutants bias developing cortical interneurons towards those that are somatostatin positive. The increase in somatostatin-positive interneurons could also be prevented by pharmacological inhibition of the core RAS/MAPK signaling pathway. Overall, these findings present new insights into how different RAS/MAPK mutations can converge on GABAergic interneurons, which may be important for other RAS/MAPK genes and related disorders.

## Teaser

**Summary:** Hyperactivation of the RAS/MAPK pathway in mouse via two different mechanisms reveals that distinct RAS/MAPK genes influence the molecular and cellular properties of GABAergic interneurons, suggesting common alterations that may be relevant to the large family of RAS/MAPK syndromes.

## INTRODUCTION

Cellular signaling via the RAS/MAPK cascade is a crucial regulator of multiple cellular and molecular developmental milestones (Seger and Krebs, 1995; Sun et al., 2015; Waltereit and Weller, 2003). These signaling events translate various extracellular cues to downstream effectors in both the cytosol and the nucleus to impact cell proliferation, migration, morphology and synapse maturation/plasticity. Importantly, mutations in RAS/MAPK genes underlie a family of neurodevelopmental syndromes with an elevated risk of autism spectrum disorder (ASD) and cancer (Adviento et al., 2014; Hoshino et al., 1999; Vithayathil et al., 2018). Several animal studies have led to insights into how dysfunctional RAS/MAPK genes impact brain function (reviewed by Gutmann et al., 2012; Hebron et al., 2022; Kang and Lee, 2019). However, a more in-depth investigation of specific brain cell types at the cellular and molecular level that may underlie the cognitive symptoms is needed. Common phenotypes between these disorders could have major implications for future therapeutics.

Earlier studies examining the RAS/MAPK pathway inhibitor *Nf1* suggested that GABAergic dysfunction could be a key factor in the cognitive changes associated with RAS/MAPK disorders (Costa et al., 2002; Cui et al., 2008). Recent studies identified specific cellular and molecular consequences of RAS/MAPK hyperactivation in GABAergic cortical interneurons (CINs) (Pai et al., 2023), including the loss of parvalbumin (PV; also known as PVALB)^+^ CINs and a decrease in LHX6 (Angara et al., 2020; Holter et al., 2021; Omrani et al., 2015). LHX6 is a cardinal transcription factor that is necessary for the emergence of CIN populations from the medial ganglionic eminence (MGE) (Liodis et al., 2007; Vogt et al., 2014; Zhao et al., 2008). MGE-derived CINs primarily express either PV or somatostatin (SST) (Liodis et al., 2007; Zhao et al., 2008), constitute ∼70% of forebrain CINs and are necessary players in brain microcircuit function and disease (Marín, 2012; Wonders and Anderson, 2006). A gap in knowledge is how distinct GABAergic CINs become fated to attain their unique molecular, morphological and electrophysiological signatures (Hu et al., 2017a; Lim et al., 2018; Mayer et al., 2018; Wamsley and Fishell, 2017). Whether cellular events, particularly RAS/MAPK signaling, could be involved has not been thoroughly explored. This is an important developmental question, as the PV and SST interneuron types are derived from the same progenitor cells in the embryonic MGE (Hu et al., 2017a; Wamsley and Fishell, 2017; Wonders and Anderson, 2006), yet mature into distinct cell types in mice. One hypothesis of how distinct properties arise is through engagement of activity-dependent processes as CINs integrate into their respective target locations (Close et al., 2012; De Marco García et al., 2011; Denaxa et al., 2012; Wamsley and Fishell, 2017). Given that RAS/MAPK signaling is elevated by neural activity (Adams and Sweatt, 2002; Thomas and Huganir, 2004; West et al., 2001), it is possible that activity-dependent recruitment of RAS/MAPK impacts the development of GABAergic interneurons via changes in core transcriptional programs necessary for their development. Despite these observations, no one has tested whether these observations converge in CINs.

We thus investigated whether core GABAergic and CIN developmental programs were altered in two distinct genetic animal models that lead to hyperactive RAS/MAPK signaling, building upon recent work that examined how hypofunction of the RAS/MAPK pathway impacts development (Knowles et al., 2022 preprint). Although mutations in RAS/MAPK signaling genes are implicated in cognitive changes in the RASopathies, there is substantial variability between individuals, potentially owing to their specific gene mutation and/or hierarchy of the gene product in the signaling pathway (Adviento et al., 2014). Despite these challenges, common phenotypic changes shared between different RAS/MAPK mutants may also exist and could be a fundamental inroad to treatment of overlapping symptoms in RASopathies. To uncover these features, we assessed *Nf1* loss of function and *bRaf* (*Braf*) constitutively active (ca) (hereafter *bRaf^ca^*) genetic mouse models in CINs, with the goal of identifying what common changes occur when RAS/MAPK signaling was amplified.

We uncovered RAS/MAPK-induced alterations in CINs impacting core developmental genes involved in cell fate and function. Hyperactive RAS/MAPK gene mutants resulted in a bias towards SST-expressing cells with correlative physiological properties at the expense of PV-expressing CINs. We also found that neuronal activity-induced RAS/MAPK signaling is one way in which SST-expressing CINs are selectively biased, potentially bridging several known observations about neural activity and its role in recruiting RAS/MAPK signaling (Tyssowski et al., 2018; Wiegert and Bading, 2011) as well as growth factor and activity-induced SST expression (Tolon et al., 1994; Zeytin et al., 1988). These results suggest that a common GABAergic phenotypic program is altered in hyperactive RASopathies and that RAS/MAPK signaling is one conduit for how extracellular cues/cellular signaling can influence the molecular properties of cells in the MGE.

## RESULTS

### *Nf1* and *bRaf^ca^* mutants exhibit similar decreases in PV but distinct changes to SST CINs by adult ages

We used a genetic approach to manipulate different RAS/MAPK genes, first comparing *Nf1* loss with *bRaf^ca^* mutants; each results in hyperactivation of the MAPK signaling cascade. The function and stratification of these and other RAS/MAPK proteins are shown in Fig. S1. This approach allowed us to discern phenotypes resulting from *Nf1* deletion (upstream inhibitor of the pathway), which regulates multiple signaling cascades, versus selective hyperactivation of the RAS/MAPK pathway, via downstream *bRaf* constitutive activation. Cre-dependent *bRaf^ca^* (Urosevic et al., 2011) or *Nf1* floxed mice (Zhu et al., 2001) were crossed with *Nkx2.1-Cre* (Xu et al., 2008) and *Ai14* alleles (Madisen et al., 2010) to generate wild-type (WT), *Nf1* conditional knockout (cKO) and hemizygous *bRaf^ca^* embryos that express tdTomato in Cre-recombined cells.

**Fig. 1.**
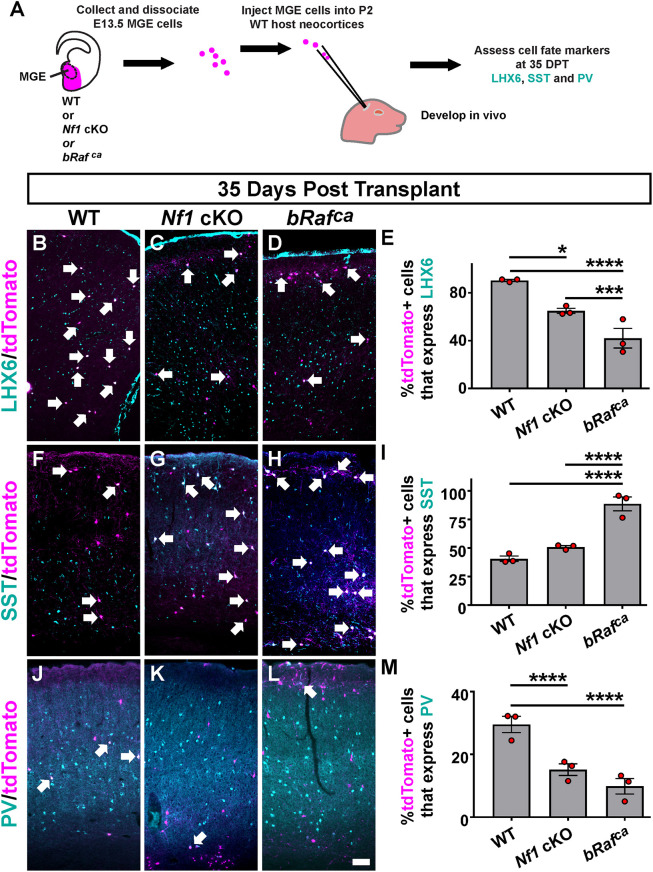
***Nf1* and *bRaf* MGE transplants reveal altered LHX6, SST and PV expression by mature ages.** (A) Schema depicting the MGE cell transplantation assay. Briefly, E13.5 MGE progenitors were harvested, dissociated and then injected into the neocortex of a WT host neonatal mouse. The cells developed and matured *in vivo* and were then assessed for molecular markers 35 days post-transplantation (DPT). Transplanted and mature WT, *Nf1* cKO or *bRaf^ca^* CINs were then assessed for the proportion of MGE-transplanted CINs expressing LHX6 (B-E), SST (F-I) or PV (J-M), revealing decreased LHX6 and PV expression in both mutant CINs and a unique increase in SST expression in the *bRaf^ca^* CINs. Arrows denote co-labeled cells. Data are expressed as mean±s.e.m., *n*=3 for each group, number of cells counted reported in Table S1. *P<0.05, ***P<0.001, ****P<0.0001 (Chi-squared test). Scale bar: 100 µm.

We first needed a way to compare these two gene manipulations in CINs of young adult mice. However, two issues had to be managed. *Nkx2.1-Cre*-induced recombination resulted in no live *bRaf^ca^* pups, precluding adult assessments, and *Nf1* mutants exhibit elevated numbers of premature oligodendrocytes (Angara et al., 2020). To navigate these obstacles, we used an MGE cell-transplantation approach that has been used to assess molecular and cellular phenotypes of mature CINs *in vivo* from mutant mice that exhibit premature lethality (Vogt et al., 2014). In addition, CINs are unique in their ability to disperse and migrate once transplanted into the brain (Alvarez-Dolado et al., 2006), allowing us to physically separate CINs from oligodendrocytes *in vivo*. To this end, embryonic day (E) 13.5 MGE cells were collected from *Nkx2.1-Cre; Ai14* embryos that were WT, *Nf1* cKO or *bRaf^ca^*, transplanted into postnatal day (P) 2 WT neocortices and allowed to develop *in vivo* for 35 days (Fig. 1A).

The transplanted cells expressed tdTomato and were co-labeled for LHX6, SST or PV (Fig. 1B-D,F-H,J-L), allowing us to assess the proportion of MGE-lineage transplanted cells that expressed each marker after their development and maturation *in vivo*. The percentages of *Nf1* cKO and *bRaf^ca^* tdTomato^+^ cells that expressed LHX6 were decreased by 28% and 50%, respectively, compared with WT, providing support that this molecular phenotype is cell autonomous and shared between the mutants (Fig. 1E; WT versus *Nf1* cKO *P*=0.04, WT versus *bRaf^ca^ P*<0.0001, *Nf1* cKO versus *bRaf^ca^ P*=0.0002). We did detect tdTomato^+^ oligodendrocytes in *Nf1* cKO transplants, but they remained at the injection site. Notably, *bRaf^ca^* mutant cells had larger somas (Fig. S2; *bRafca* versus WT and *Nf1* cKO *P*<0.0001).

We next examined the expression of SST in the transplanted cells. In agreement with our previous studies, the proportion of *Nf1* cKO cells at this mature age that expressed SST was similar to WTs (Fig. 1I) (Angara et al., 2020; Holter et al., 2021). In contrast, most of the *bRaf^ca^* cells expressed SST at high levels (Fig. 1I; WT and *Nf1* cKO versus *bRaf^ca^ P*<0.0001). Finally, we determined the proportion of transplanted cells that expressed PV. Both the *Nf1* cKOs and *bRaf^ca^* mutants had decreased expression of PV, by 48% and 70%, respectively (Fig. 1M; WT versus *Nf1* cKO and *bRaf^ca^ P*<0.0001). Overall, each mutant exhibited alterations in CIN markers with the more pronounced phenotypes observed in *bRaf^ca^* mutants.

### Postmitotic depletion of *Nf1* leads to a reduction in LHX6 and the SST/PV ratio

We next tested whether the loss of LHX6 was due to alteration in MGE progenitor cells or if this was a postmitotic phenomenon. To this end, we crossed both *Nf1^Flox^* and *bRaf^ca^* mice to *Lhx6-Cre* mice, to deplete the genes at a later developmental stage, as cells are becoming postmitotic. Unfortunately, we were not able to collect live *Nf1* cKO or *bRaf^ca^* progeny at postnatal stages, likely owing to *Lhx6-Cre* recombination in blood vessels (Fogarty et al., 2007). However, we acquired viable *Nf1* conditional heterozygous (cHet) mice, which survived to P30, to assess LHX6 protein expression. We found a ∼47% reduction of LHX6 expression in *Lhx6-Cre; Nf1* cHets compared with WTs (Fig. S3; *P*=0.004). These data indicate that reduced *Nf1* in postmitotic neurons can suppress LHX6 expression and this phenotype is not due to disruption of progenitor MGE cell biology.

**Fig. 2.**
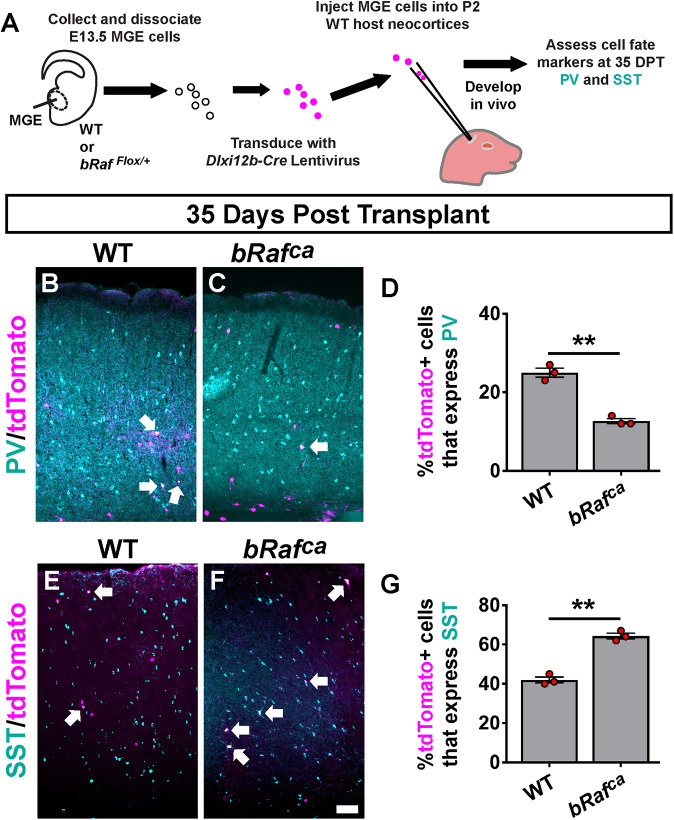
**Delayed Cre expression in postmitotic CINs results in elevated SST and decreased PV levels.** (A) Schema showing the MGE transplant approach utilizing *Dlxi1/2b-Cre* to activate *bRaf^ca^*. (B-G) WT and *bRaf^ca^* CINs at 35 days post-transplantation (DPT) were labeled for PV (B,C) or SST (E,F); arrows point to co-labeled cells. Quantification revealed a decrease in PV^+^ cells (D) and an increase in SST^+^ cells (G). Data are expressed as mean±s.e.m., *n*=3 for each group, number of cells counted reported in Table S1. **P<0.01 (Chi-squared test). Scale bar: 100 µm.

To determine whether other phenotypes could arise in these mutants in postmitotic CINs, we performed similar MGE transplants, except using lentivirus to drive *Cre* instead of the *Nkx2.1-Cre* line (Fig. 2A). Cre expression was under the control of the *Dlxi1/2b* enhancer (Vogt et al., 2015b), which biases expression to GABAergic neurons; these cells would be postmitotic. Transplanted cells were examined at 35 days post-transplantation for PV and SST. Consistent with *Nkx2.1-Cre* phenotypes, we found that lentiviral Cre resulted in similar decreased PV and increased SST levels (Fig. 2B-G; PV *P*=0.002, SST *P*=0.002). Thus, these data suggest that delayed onset of hyperactive RAS/MAPK activity in postmitotic CINs can impact the SST/PV ratio. Subsequent data sets only assess *Nkx2.1-Cre* lineages.

### *bRaf^ca^* mutant CINs exhibit a reduction in action potential spiking kinetics

**Fig. 3.**
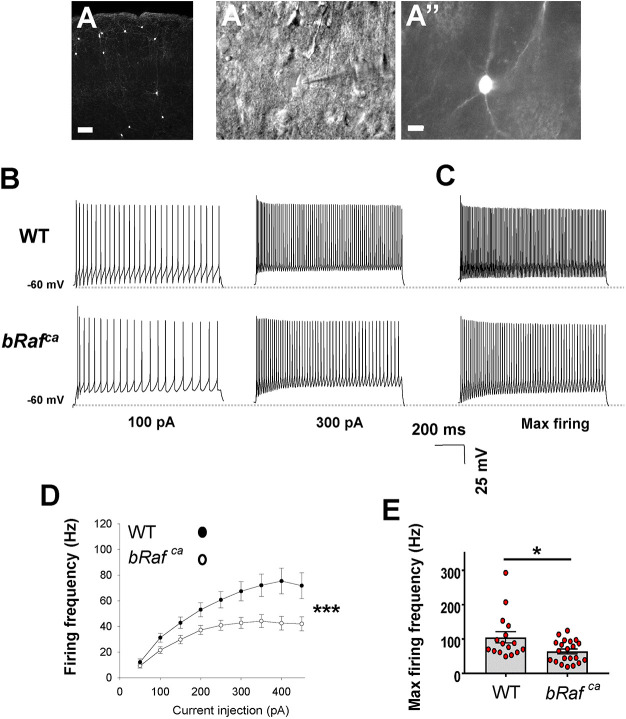
***bRaf^ca^* mutant CINs exhibit reduced action potential firing frequency.** (A,A″) Representative images showing the S1 region of the cortex with tdTomato^+^ transplanted CINs. (A′) Differential interference contrast image of a patched CIN. (B) Representative traces showing neuronal firing in response to 100 and 300 pA current injections in WT (top) and *bRaf^ca^* (bottom). (C) Representative traces during maximal firing. (D) Quantification of evoked firing frequency in WT and *bRaf^ca^* CINs at different current injections. Two-way, repeated-measures ANOVA revealed a significant effect of current and genotype [*F* (1, 53)=4.12; ***P<0.001, two-way repeated measures ANOVA with Holm–Sidak test]. (E) Quantitative analysis of maximal firing frequency between WT and *bRaf^ca^* CINs. Data are presented as mean±s.e.m. *P<0.05 (two-tailed *t*-test). The horizontal dotted line in the traces indicates −60 mV. Scale bars: 100 µm (A); 10 µm (A″).

The elevated ratio of SST^+^ to PV^+^ CINs in *bRaf^ca^* mutants (Figs 1 and 2) suggested that these mutants may exhibit a shift in CIN properties towards a SST-like CIN at the expense of the PV group. SST^+^ and PV^+^ CINs have distinct electrophysiological properties. SST^+^ CINs are mostly regular spiking and exhibit spike amplitude adaptation over time, whereas putative PV^+^ CINs are fast spiking with little to no adaptation (Halabisky et al., 2006; Hu et al., 2014; Kepecs and Fishell, 2014). Thus, if hyperactive *bRaf* resulted in a shift in cells with more SST-like properties, we hypothesized that, as a group, a loss of faster spiking properties would arise in transplanted cells. Current clamp recordings were performed in layer 2/3 of the S1 neocortex to measure spontaneous and evoked activity; example transplanted cells are shown in Fig. 3A-A″.

We assessed whether action potential spiking was different between WT and *bRaf^ca^* groups of transplanted cells from *Nkx2.1-Cre* lineages. Example traces of spiking are shown for 100 pA and 300 pA current injections between genotypes (Fig. 3B) as well as during maximum firing (Fig. 3C). Consistent with our hypothesis, a two-way, repeated-measures ANOVA revealed a significant effect of current and genotype (Fig. 3D) [*F* (1, 53)=4.12; *P*<0.001; Holm–Sidak test]; action potential amplitude for both groups was similar. Finally, maximum evoked spike frequency was significantly reduced in *bRaf^ca^* CINs (Fig. 3E; *P*=0.01). These data support that *bRaf^ca^* mutants can promote CIN electrophysiological properties towards lower action potential spiking frequencies.

We also assessed passive and active properties of the transplanted CINs (Table S2). Many properties were not significantly changed, including membrane capacitance, resting membrane potential, as well as resting and active membrane resistance. Importantly, mutant CINs mostly resembled WT cells, suggesting proper maturation. Consistent with the decreased maximum firing frequency, we also noticed increased interspike interval (ISI) length in the mutant cells. Mutants exhibited a longer initial ISI (*P*=0.04). Although the last ISI was ∼28% longer in the mutants it did not reach significance. In addition, mutant CINs were slower to elicit a first action potential following a 400 pA pulse, suggesting delayed kinetics. Overall, *bRaf^ca^* mutant CINs have shifted dynamics that are more aligned with SST-like CINs, but may not exhibit a full shift in properties towards this group.

### Elevated SST expression is a common hyperactive RAS/MAPK phenotype

**Fig. 4.**
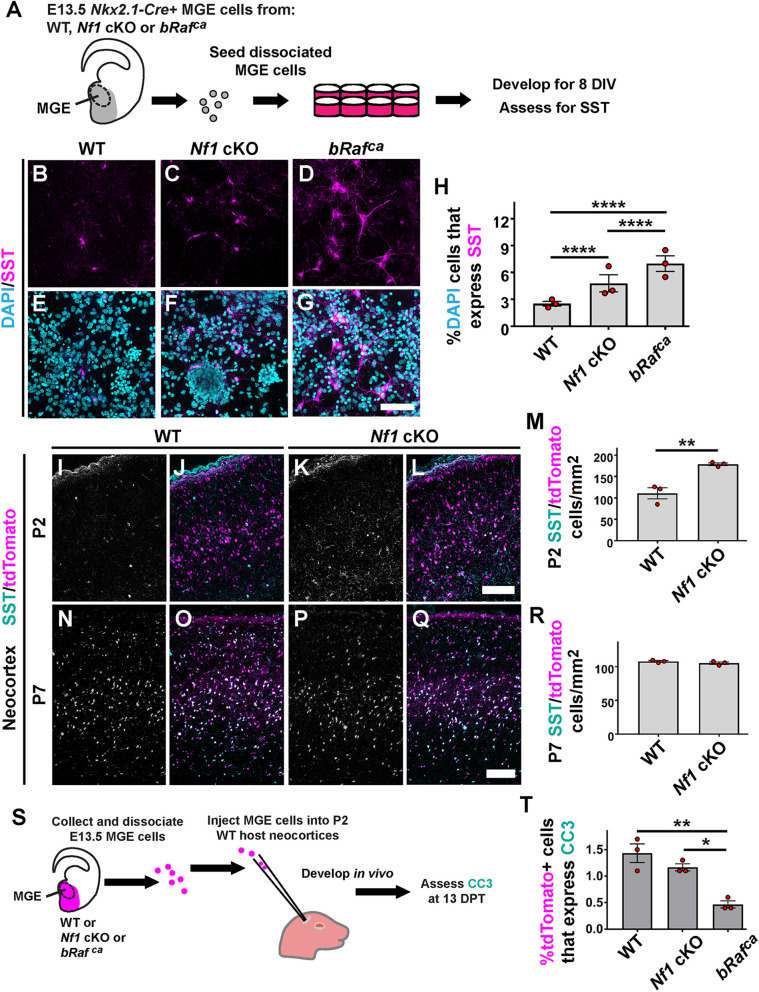
**Elevated SST CINs are a common phenotype of *Nf1* and *bRaf* mutants in early development.** (A) Schema depicting MGE primary culture procedures (gray area depicts *Nkx2.1-Cre* domain); E13.5 MGE progenitors were dissociated and grown for 8 days *in vitro* (DIV) before assessing SST levels. (B-G) Images of SST- and DAPI-stained primary cultures after 8 DIV. (H) Quantification of the proportion of DAPI^+^ cells expressing SST; elevated SST numbers were found in both mutants (Chi-squared test). (I-L,N-Q) Neocortical images of CINs (tdTomato^+^) co-labeled for SST. (M,R) Quantification of tdTomato^+^/SST^+^ cells at P2 and P7, respectively, showing elevated levels at P2 that normalize by P7 (two-tailed *t*-test). (S) Schema showing the cell transplant assay used to assess apoptosis. DPT, days post-transplantation. (T) Quantification of the proportion of transplanted cells that co-label for the apoptosis marker cleaved caspase 3 (CC3) (one-way ANOVA with Tukey post-hoc test). Data are expressed as mean±s.e.m., *n*=3 for each group. *P<0.05, **P<0.01, ****P<0.0001. Scale bars: 50 µm (G); 100 µm (L,Q).

To assess whether elevated SST levels and/or numbers of cells are a common phenotype in hyperactive RAS/MAPK mutants, we first assayed SST protein in MGE primary neuronal cultures from E13.5 brains, aged 8 days *in vitro* (Fig. 4A). Both *Nf1* cKO and *bRaf^ca^* cultures exhibited an elevated percentage of SST^+^ CINs (Fig. 4B-H; *P*<0.0001). Qualitative increases in total SST that filled *bRaf^ca^* mutant cells were also noted (Fig. 4D), suggesting that SST protein expression is a shared feature of elevated RAS/MAPK activity.

We also examined SST expression at early postnatal stages to determine whether the *Nf1* cKOs exhibited elevated SST expression *in vivo*. The previous primary culture experiments were aged *in vitro* to an equivalent age of P2; thus, we assessed SST levels at P2 in the neocortex and found an ∼62% increase in *Nf1* cKO CINs expressing SST (Fig. 4I-M; *P*=0.007), consistent with the primary cultures. By P7, there was no difference in SST expression between WTs and *Nf1* cKOs (Fig. 4N-R). Because both the *Nf1* cKO and *bRaf^ca^* embryos had elevated SST^+^ levels without changes in total tdTomato^+^ CINs (S.J.K. and J.M.N., unpublished observations; Angara et al., 2020), we first concluded that hyperactive MAPK mutants have a developmental preference to bias MGE towards SST^+^ CINs.

The early developmental preference in the mutants to bias SST^+^ over PV^+^ CINs could explain the deficit in PV^+^ CINs at more mature ages. However, there are some discrepancies between different mutations; *bRaf^ca^* mutant CINs had elevated SST^+^ numbers but *Nf1* cKOs had normal levels at adult stages. The developmental stage between P2 and P7 for CINs is marked by programmed apoptosis (Southwell et al., 2012). Because RAS/MAPK signaling promotes cell survival (Bonni et al., 1999), we examined whether hyperactive MAPK mutations altered cell death. We allowed WT, *Nf1* cKO and *bRaf^ca^* MGE transplants to develop for 13 days post-transplantation, and assessed CIN cell death during the peak apoptosis window (Southwell et al., 2012). We found less apoptosis in *bRaf^ca^* mutants (Fig. 4S,T; *bRaf^ca^* versus WT *P*=0.006, *bRaf^ca^* versus *Nf1* cKO *P*=0.045). Thus, although *Nf1* cKO and *bRaf^ca^* CINs each exhibit increased SST expression early, the *bRaf^ca^* CINs partially elude programmed apoptosis during development, resulting in the same loss of PV but differential SST ratios in mature cells.

### *Nf1* and *bRaf^ca^* mutations have unique and common effects on core MGE proteins

**Fig. 5.**
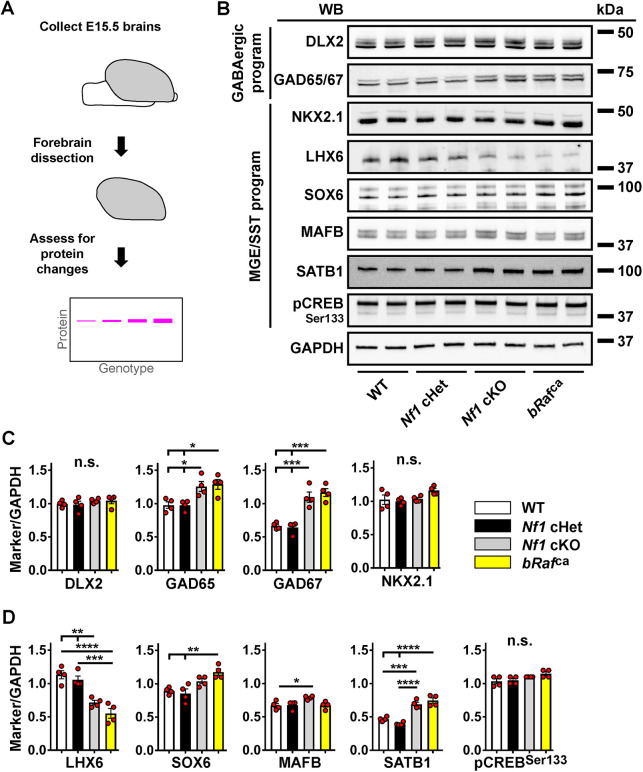
**Biochemical assessment of GABAergic and MGE-lineage genes.** (A) Schema to depict the collection of forebrain tissue for western blot analyses from E15.5 forebrains. (B) Western blots from representative pairs of genotypes probed for GABAergic program proteins and more-specific MGE/SST program proteins; SOX6 is the middle band in the image. GAPDH was used as a loading control. (C) Quantification of protein of interest band intensity divided by GAPDH band intensity for GABAergic and patterning markers. (D) Quantification of band intensities for more specific MGE/SST program markers. Data are expressed as mean±s.e.m., *n*=4 for each group. *P<0.05, **P<0.01, ***P<0.001, ****P<0.0001 (one-way ANOVA with Tukey post-hoc test). n.s., not significant; WB, western blot.

CIN development is regulated by well-defined transcription factors, although how these programs are influenced by MAPK signaling is largely unknown. Thus, we investigated whether *Nf1* cKO and *bRaf^ca^* mutants have altered core GABAergic and MGE-lineage programs in the embryonic forebrain. To this end, we focused on proteins involved in these programs in either *Nf1* cHets or cKOs as well as *bRaf^ca^* embryos. We chose E15.5 for assessment, as brains at this age have MGE-derived cells that are undergoing multiple developmental milestones, including continued propagation and migration throughout the cortex. Dissection of the brain (Fig. 5A) was performed to remove hindbrain/midbrain structures while preserving forebrain.

Western blots for candidate proteins involved in the broad GABAergic program [DLX2 and GAD65/67 (GAD2/1)] or MGE patterning (NKX2-1) were performed (Fig. 5B). DLX2 and NKX2-1 levels were unchanged (Fig. 5B,C). GAD65/67 levels were increased in *Nf1* cKO and *bRaf^ca^* brains (Fig. 5B,C; GAD65: WT and *Nf1* cHet versus *Nf1* cKO *P*=0.3, WT and *Nf1* cHet versus *bRaf^ca^ P*=0.02; GAD67: WT versus *Nf1* cKO *P*=0.0006, WT versus *bRaf^ca^ P*=0.0002, *Nf1* cHet versus *Nf1* cKO *P*=0.0004, *Nf1* cHet versus *bRaf^ca^ P*=0.0001), suggesting a role for MAPK activity in the activity-dependent regulation of GAD genes (Hanno-Iijima et al., 2015). Because we used whole forebrain, the increase in GAD proteins could occur in ventral and/or dorsal regions, the latter containing most migrating CINs. We thus stained E15.5 forebrain tissue for GAD67 and found that, whereas no gross elevation in protein was seen in dorsal regions, ventral domains had elevated GAD67 expression (Fig. S4; WT versus *Nf1* cKO *P*=0.0003, WT versus *bRaf^ca^ P*<0.0001). Because dorsal regions were unchanged, we did not pursue analyses of the GAD proteins further.

### LHX6 is commonly downregulated in *Nf1* cKO and *bRaf^ca^* mutants

As expected, LHX6 protein was decreased in both *Nf1* cKOs and *bRaf^ca^* brains (Fig. 5B,D; WT versus *Nf1* cKO *P*=0.001, WT versus *bRaf^ca^ P*<0.0001, *Nf1* cHet versus *Nf1* cKO *P*=0.006, *Nf1* cHet versus *bRaf^ca^ P*=0.0002); levels in *Nf1* cHets decreased at later ages (Fig. S3; Angara et al., 2020). Additionally, we assessed E15.5 *bRaf^ca^* brains for LHX6 protein expression in *Nkx2.1-Cre*-lineage cells. Although the cell density of *Nkx2.1-Cre*-lineage cells (tdTomato^+^) was not altered between genotypes (Fig. S5A,D,G,J,M), the proportion of tdTomato^+^ cells that co-labeled for LHX6 protein were only approximately half as numerous in *bRaf^ca^* brains compared with littermate controls in the neocortex (Fig. S5B,C,E,F,H,I,K,L,N; *P*<0.0001). Thus, *bRaf^ca^* mutants exhibit an early loss of LHX6, more severe than that of *Nf1* cHet and cKO mutants.

### SATB1 is commonly upregulated in *Nf1* cKO and *bRaf^ca^* mutants

LHX6 can modulate the expression of several genes that may underlie SST expression in the mutants. To this end, we examined three markers known to be involved in the promotion of SST cell fate: SOX6, MAFB and SATB1 (Close et al., 2012; Denaxa et al., 2012; Hu et al., 2017b; Pai et al., 2019; Vogt et al., 2014). Mildly elevated MAFB protein was found in *Nf1* cKOs (Fig. 5B,D; *P*=0.04), but not *bRaf^ca^* samples, suggesting a potential unique role for *Nf1* in the control of this MGE-lineage gene. SOX6 also had elevated expression within the *bRaf*, but not *Nf1*, mutants (Fig. 5B,D; WT versus *bRaf^ca^ P*=0.005, *Nf1* cHet versus *bRaf^ca^ P*=0.002). Surprisingly, pCREB was not altered in the mutants (Fig. 5B,D), despite reported positive regulation by RAS/MAPK signaling and its ability to directly transduce SST (Gonzalez and Montminy, 1989; Wu et al., 2001). However, the most striking change was the increase in SATB1 levels in both *Nf1* cKOs and *bRaf* mutants (Fig. 5B,D; WT versus *Nf1* cKO *P*=0.0004, WT and *Nf1* cHet versus *bRaf^ca^ P*<0.0001, *Nf1* Het versus *Nf1* cKO *P*<0.0001). Because SATB1 overexpression can lead to an increase in SST expression, and SATB1 can directly bind to the SST promoter (Balamotis et al., 2012; Denaxa et al., 2012; Goolam and Zernicka-Goetz, 2017; Tu et al., 2019), SATB1 is a candidate for the elevated SST levels.

### SATB1 expression is elevated in *Nkx2.1-Cre*-lineage *Nf1* cKO and *bRaf^ca^* cells during development

**Fig. 6.**
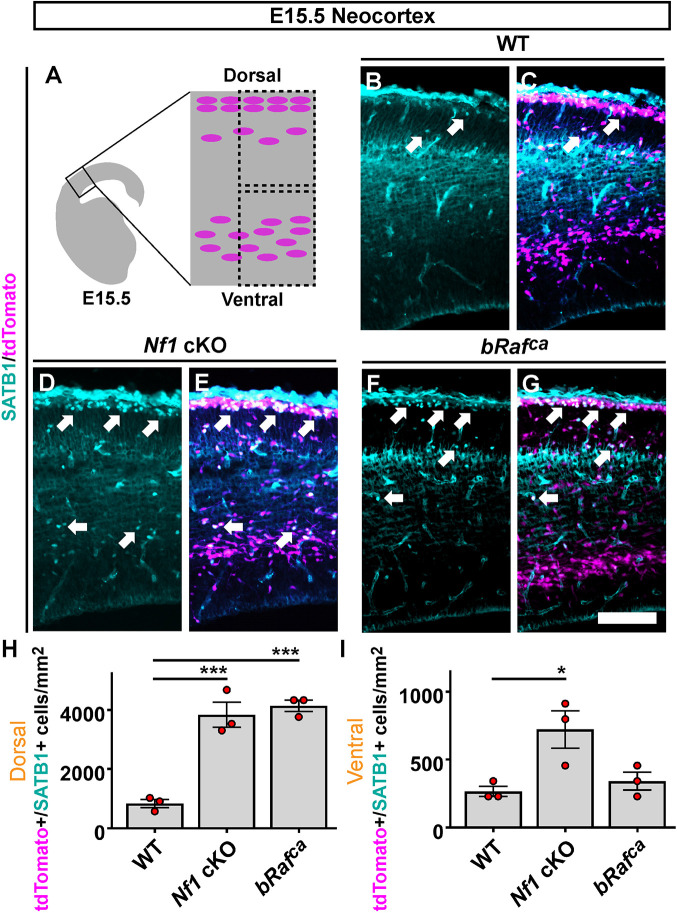
**SATB1 is aberrantly elevated in *Nkx2.1-Cre* lineage CINs during embryonic development.** (A) Schema depicting the developing neocortex and dorsal/ventral regions used for assessments (dashed boxes). (B-G) Images of SATB1 protein co-labeled with tdTomato (*Nkx2.1-Cre* lineages) in the developing neocortex; arrows point to co-labeled cells. (H,I) Quantification of the number of SATB1^+^/tdTomato^+^ cells per area in dorsal (H) and ventral (I) regions; elevated SATB1 numbers were found in both mutants. Data are expressed as mean±s.e.m., *n*=3 for each group. *P<0.05, ***P<0.001 (one-way ANOVA with Tukey post-hoc test). Scale bar: 100 µm.

Although many of the factors probed by western blot are MGE derived and of the interneuron lineage at E15.5, whole forebrain was used. To validate that SATB1 protein was increased in developing CINs of the neocortex, we stained E15.5 for SATB1 and found that the number of migrating tdTomato^+^ CINs in the neocortex expressing SATB1 was increased primarily in dorsal migratory streams in the *Nf1* cKO and *bRaf^ca^* mutants. *Nf1* cKOs also had elevated SATB1^+^ cells in ventral streams, suggesting some deviation of phenotypes (Fig. 6; dorsal WT versus *Nf1* cKO *P*=0.0007, dorsal WT versus *bRaf^ca^ P*=0.0004, ventral WT versus *Nf1* cKO *P*=0.03). These results suggest that increased SATB1 in CINs derived from the MGE may be a contributor to the cell fate bias of SST^+^ CINs in hyperactive RAS/MAPK mutants. Moreover, it validates that MGE-derived migrating interneurons in the neocortex contribute to this phenotype.

### ARX is decreased in both *Nf1* cKOs and *bRaf^ca^* mutants

**Fig. 7.**
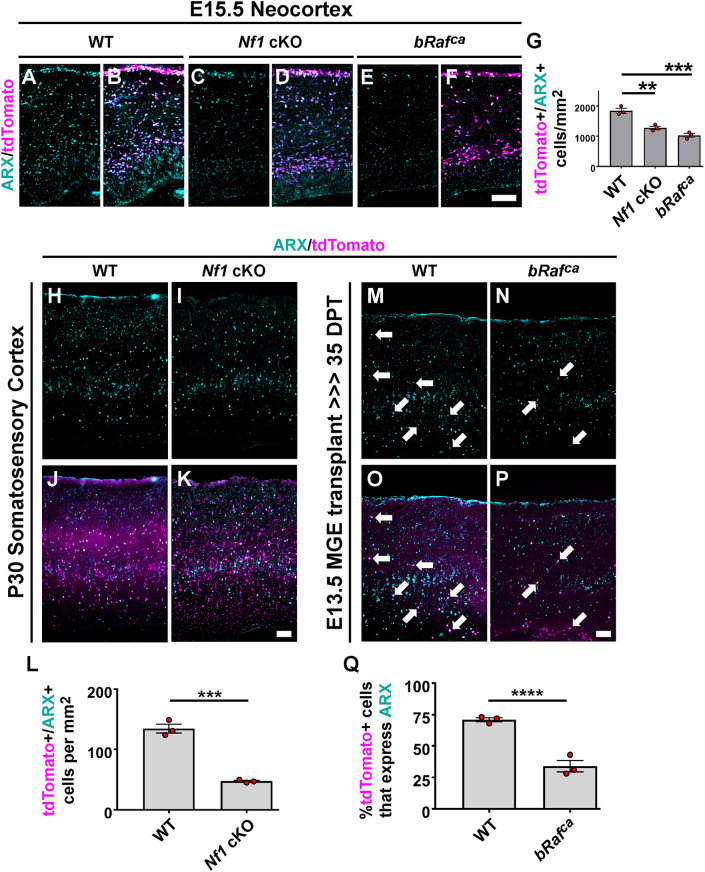
**ARX is decreased in both *Nf1* cKO and *bRaf^ca^* mutants.** (A-G) E15.5 neocortices were labeled for ARX protein (A-F); quantification revealed a decrease in the cell density of CINs (tdTomato^+^) expressing ARX in both *Nf1* cKO and *bRaf^ca^* brains (G) (one-way ANOVA with Tukey post-hoc test). (H-L) P30 cortices were labeled for ARX expression in WT and *Nf1* cKOs at P30 (H-K), revealing a decrease in *Nf1* cKO ARX-labeled CINs (L) (two-tailed *t*-test). (M-Q) WT and *bRaf^ca^* E13.5 MGE transplants aged to 35 days post-transplantation (DPT) were also labeled for ARX (M-P); arrows point to co-labeled cells. *bRaf^ca^* transplants also showed a reduction in ARX-expressing CINs (Q) (Chi-squared test). Data are expressed as mean±s.e.m., *n*=3 biological replicates, all groups. **P<0.01, ***P<0.001, ****P<0.0001. Scale bars: 100 µm.

We also assessed whether other core GABAergic CIN programs were altered in *Nf1* cKO and *bRaf^ca^* mutants. The aristaless homeobox *Arx* gene is one such factor, but because of high expression in other cell types its protein product could not be assessed reliably by western blotting. In addition to being regulated by LHX6 and DLX proteins (Colasante et al., 2008; Vogt et al., 2014; Zhao et al., 2008), ARX it also controls CIN developmental properties (Friocourt et al., 2008; Joseph et al., 2021; Marsh et al., 2016; Ruggieri et al., 2010). We examined E15.5 brains for ARX expression and found 31% and 44% reductions in *Nf1* cKO and *bRaf^ca^* brains, respectively (Fig. 7A-G; WT versus *Nf1* cKO *P*=0.003, WT versus *bRaf^ca^ P*=0.0004). To determine whether the loss of ARX persisted in mature CINs, we first examined ARX expression in somatosensory cortices of WT and *Nf1* cKO P30 brains. ARX expression was decreased by 65% in *Nf1* cKO CINs (Fig. 7H-L; *P*=0.0003). We also assessed an equivalent age for WT and *bRaf^ca^* MGE-transplanted cells. Consistent with earlier data, the proportion of transplanted CINs expressing ARX was reduced by 52% (Fig. 7M-Q; *P*<0.0001). Thus, ARX reduction is another shared phenotype between these two hyperactive MAPK mutants.

### Pharmacological blockade of MEK signaling normalizes SST expression in hyperactive RAS/MAPK mutants

**Fig. 8.**
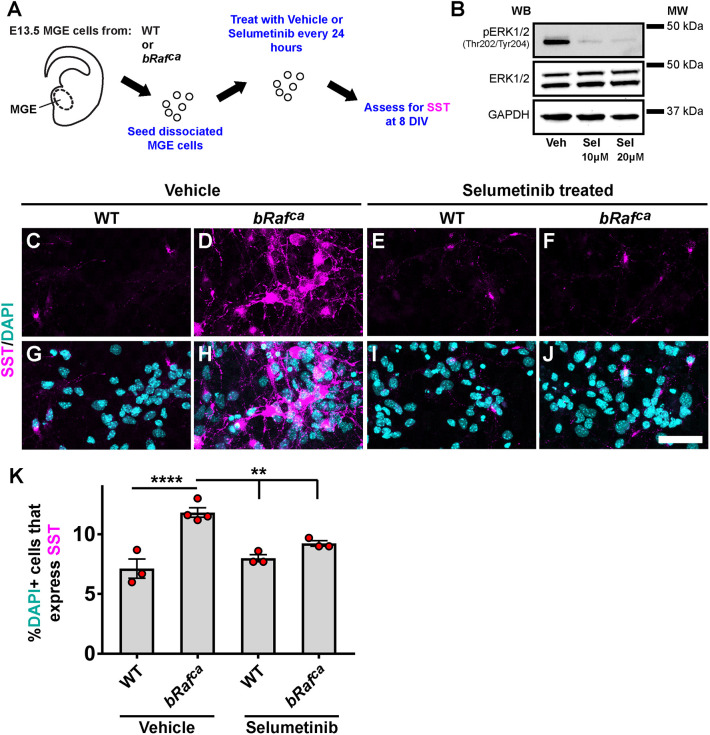
**MEK inhibition prevents elevated SST expression in *bRaf^ca^* mutants.** (A) Schema depicting the paradigm. E13.5 MGE cells were collected, dissociated and cultured in the presence of vehicle or selumetinib for 7 days *in vitro* (DIV). (B) Western blots of WT cells cultured in either vehicle (Veh) or drug (Sel) were probed for pERK, total ERK and GAPDH at 7 DIV; a 20 µM dose of drug was chosen for subsequent use. MW, molecular weight. (C-J) Images of primary cultures labeled for SST and DAPI at 7 DIV showing elevated SST expression in the *bRaf^ca^* mutant that is prevented by drug treatment. (K) Quantification of the proportion of DAPI^+^ cells expressing SST. Data are expressed as mean±s.e.m., *n*=3-4 for each group. **P<0.01, ****P<0.0001 (Chi-squared test). Scale bar: 50 µm.

The increase in SST^+^ CINs across these two distinct models suggested a link between MAPK signaling and SST expression. To test this, we employed the recently FDA-approved drug selumetinib, a more specific MEK inhibitor that can cross the blood–brain barrier (Liang et al., 2018; McNeill et al., 2017; Van Swearingen et al., 2017). MEK activity is downstream of the proteins encoded by both *Nf1* and *bRaf* (Fig. S1). To test whether selumetinib could normalize SST expression, we generated MGE primary cultures from WT or *bRaf^ca^* brains and treated with either vehicle or drug every 24 h for 8 days before assessing SST expression (Fig. 8A). Western blots of WT cultures treated with vehicle or 10 µM or 20 µM of selumetinib were assessed for pERK to determine efficacy (Fig. 8B). Both drug doses were effective at reducing pERK levels; the 20 µM dose was used for subsequent experiments.

As expected, in vehicle-treated cultures, elevated SST levels were observed in *bRaf^ca^* CINs (Fig. 8D,F,H,J,K; *P*<0.0001). Treatment with 20 µM selumetinib led to an attenuation of SST levels in the *bRaf^ca^* mutants but did not alter WT levels (Fig. 8C,E,G,I,K; vehicle WT versus vehicle *bRaf*^*ca*^ *P*<0.0001, vehicle *bRaf*^*ca*^ versus selumetinib WT *P*=0.008, vehicle *bRaf*^*ca*^ versus selumetinib *bRaf*^*ca*^ *P*=0.009). Thus, the increase in SST expression is dependent upon MAPK signaling.

**Fig. 9.**
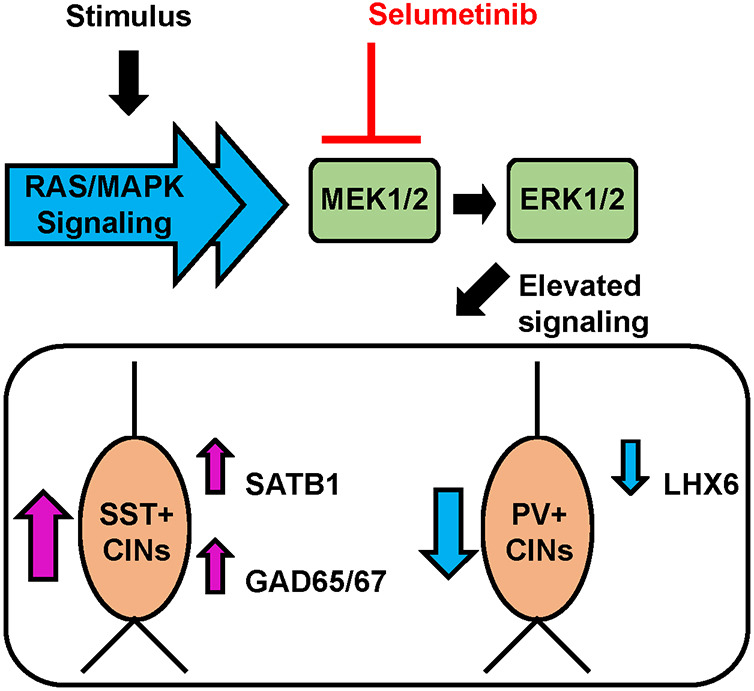
**Multiple stimuli can recruit RAS/MAPK activity to transduce signals throughout a cell.** Although some RAS/MAPK proteins can signal through a variety of pathways, a shared core MAPK signaling pathway exists, which utilizes MEK1/2 (MAP2K1/MAP2K2) and ERK1/2 (MAPK3/1) proteins. We found that RAS/MAPK hyperactive mutants also exhibited unique and common effects on GABAergic cortical interneurons. Specifically, the bias in producing SST over PV CINs and common molecular phenotypes, including the increase in SATB1 and GAD65/67 proteins with concomitant decrease in LHX6. Our data show that these newly described phenotypes can be attenuated with the MEK inhibitor selumetinib, suggesting that these phenotypes are RAS/MAPK signaling dependent. These data provide an important role for canonical RAS/MAPK signaling events that impinge upon core GABAergic CIN programs.

These data suggest that the elevation in SST levels is dependent on RAS/MAPK signaling and provide a potential mechanism for how events that activate MAPK signaling could induce SST^+^ CIN properties via a powerful signaling pathway that connects extracellular cues to potential cellular functions (Fig. 9).

## DISCUSSION

We uncovered common GABAergic CIN phenotypes caused by distinct RAS/MAPK hyperactive gene mutations. Some of these phenotypes are due to hyperactivation of the core RAS/MAPK signaling pathway. Seminal studies have pointed to the role of cardinal transcription factors in guiding interneuron cell fate and function (Liodis et al., 2007; Long et al., 2009; Sussel et al., 1999; Vogt et al., 2014; Zhao et al., 2008). Recently, neural activity and cell signaling have also emerged as important factors that guide GABAergic interneuron development and maturation (Close et al., 2012; De Marco García et al., 2011; Denaxa et al., 2012; Malik et al., 2019; McKenzie et al., 2019; Vogt et al., 2015b; Wundrach et al., 2020). Recruitment of RAS/MAPK signaling and induction of SST expression (Tolon et al., 1994; Tyssowski et al., 2018; Wiegert and Bading, 2011; Zeytin et al., 1988) make RAS/MAPK signaling an interesting potential candidate as a mechanism to influence CIN development downstream of a myriad of extracellular cues as studies in cellular signaling upon CINs emerge (Pai et al., 2023). Our data suggest that MGE cells may bias towards SST CINs via activation of RAS/MAPK signaling, recently supported by RAS/MAPK loss-of-function studies (Knowles et al., 2022 preprint).

CIN development and maturation follow a well-studied timeline to produce unique cellular and molecular properties in CIN classes (Hu et al., 2017a; Lim et al., 2018; Mayer et al., 2018; Wamsley and Fishell, 2017; Wonders and Anderson, 2006). During mid gestation, CINs are primarily generated in the MGE and caudal ganglionic eminence of the ventral telencephalon and, after becoming post-mitotic, begin a long migration to their final destinations that can be influenced by local cues and the dynamic structure of the developing brain (Fazzari et al., 2020; Wonders and Anderson, 2006). Our experiments sought to determine when phenotypes may be induced, i.e. in progenitors and/or postmitotic CINs. During these processes, cellular and molecular properties start to diverge in different CIN cell types before the CINs find their synaptic partners and form the unique microcircuits of the cortex. Although delayed lentiviral expression of Cre and the GABAergic-biased *Dlxi1/2b* enhancer have been used in combination to assess whether phenotypes could occur in postmitotic cells as well, it is possible that this approach may not be completely restricted to postmitotic CINs. Studies have elucidated core transcription factors involved in these processes as well as the role of neural activity in these events (Batista-Brito et al., 2009; Butt et al., 2008; Close et al., 2012; Denaxa et al., 2012; Marsh et al., 2016; Pai et al., 2020; Pla et al., 2018). We found that distinct hyperactive RAS/MAPK mutants had common changes in some core GABAergic programs. These distinct mutants included biallelic loss of *Nf1* and a constitutively active *bRaf* allele, each leading to hyperactivation of the pathway but in unique ways. Importantly, the loss of *Nf1* eliminated an inhibitor of the pathway, whereas the *bRaf* allele constantly drove activation of the pathway. This is important because normally the pathway is not being activated all the time and therefore this approach could reveal insights into what hyperactive mutants could induce versus developmental events that could change as a result of normal bouts of RAS/MAPK signaling. By studying both the similarities and disparities between the mutants, new insights could be gained in normal brain development, RAS/MAPK syndromes and timing/dosage of the pathway.

We found common changes in core proteins that direct development of CINs, including LHX6, SATB1 and ARX. LHX6 is an early determinant of MGE cell fate that is necessary for the emergence of SST and PV CINs (Liodis et al., 2007; Zhao et al., 2008) and promotes the expression of SATB1 and ARX (Denaxa et al., 2012; Zhao et al., 2008). Although the loss of ARX may be a result of the depletion of LHX6 herein, it seems unlikely that this is the case for SATB1 as expression increased in the mutants, suggesting an alternative route of SATB1 gene or protein regulation in CINs. SATB1 is a likely candidate for the increase in SST expression in the hyperactive mutants, as previous data have shown expression of SATB1 is sufficient to induce SST expression in MGE lineages, even in *Lhx6* loss-of-function mutants (Denaxa et al., 2012). Other core programs were not commonly altered in the hyperactive mutants, suggesting some selectivity in CIN programs regulated by RAS/MAPK activity. Interestingly, SST^+^ CINs may be favored over PV^+^ in *bRaf^ca^* mutants as a result of reduced programmed apoptosis; the loss of another syndromic protein, PTEN, using similar genetic strategies, led to a reduction of SST^+^ CIN numbers (Vogt et al., 2015b). Although our data suggest that increased RAS/MAPK activity in early postmitotic CINs promotes a greater number of SST^+^ over PV^+^ CINs, this is not likely to be the only way SST-like cell properties could be attained, as other research has shown that premature exit from the cell cycle in the MGE can favor SST^+^ CINs (Petros et al., 2015). Future studies are needed to understand the full breadth of these changes and the impact of RAS/MAPK activity on these crucial cell types during distinct developmental stages.

Our data provide compelling evidence for a role of RAS/MAPK signaling in the development of CINs. The core GABAergic CIN changes noted above do seem to be common events in the RASopathy models studied here and we predict other RASopathy models could benefit from these findings. Those RASopathy genes with ubiquitous or enriched GABAergic expression compared with excitatory cells (Ryu et al., 2019), including *Hras*, *Kras*, *Mapk1*, *Ptpn11*, *Sos1* and *Spred1*, may be of particular relevance. In turn, if common phenotypes continue to be found in additional RAS/MAPK mutants, it could also imply that shared co-morbid symptoms, for example in attention deficit hyperactivity disorder, ASD and learning deficits, may be potentially treated in future studies by manipulation of GABAergic neurons.

## MATERIALS AND METHODS

### Animals

All mouse lines used have been described previously. We bred *Nkx2.1-Cre* mice (Xu et al., 2008) with either *Nf1^Flox^* (Zhu et al., 2001) or *bRaf^Flox-V600E^* knock-in mice (Urosevic et al., 2011), which express constitutively active *bRaf^V600E^* after Cre-recombination. Crosses included the *Ai14* (Madisen et al., 2010) Cre-dependent reporter, which drives tdTomato expression. *bRaf* mutant mice were initially on a C57BL/6 background and were backcrossed to CD-1 for at least three generations before experiments, to better match the genetic background of the *Nf1* mutants previously analyzed (Angara et al., 2020). *Lhx6-Cre* (Fogarty et al., 2007) mice have been previously described. *Lhx6-Cre* mice were crossed to *Nf1^Flox^* and Cre expression begins as MGE cells become postmitotic in the MGE. In all conditions, males and females were compared but we did not find gross differences between sexes for phenotypes; biological replicates are a combination of both sexes. Experiments were approved by Michigan State University's Campus Animal Resources and the Institutional Animal Care and Use Committee at Arizona State University.

### Electrophysiology

Mice (postnatal age 6-7 weeks) were anesthetized with 500 µl of tribromoethanol (Avertin) and coronal brain slices generated in carbogen-equilibrated, ice-cold slicing solution containing (in mM): 110 C_5_H_14_ClNO, 7 MgCl_2_.6H_2_O, 2.5 KCl, 1.25 NaH_2_PO_4_, 25 NaHCO_3_, 0.5 CaCl_2_.2H_2_O, 10 glucose and 1.3 sodium ascorbate. From rostral to caudal, 250 µm-thick brain slices containing the S1 region of the cortex were cut using a vibratome (Leica VT1200) and incubated in solution (in mM): 125 NaCl, 25 NaHCO_3_, 1.25 NaH_2_PO_4_, 2.5 KCl, 1 MgCl_2_.6H_2_O, 1 CaCl_2_.2H_2_O and 10 glucose. Incubation was performed at 34°C for 1 h before recording (Zaman et al., 2011). Recordings from transplanted cells were restricted to neocortical layers 2/3 for consistency.

In K^+^-based whole-cell current clamp mode, spontaneous and evoked firing properties were recorded in tdTomato^+^ *Nkx2.1-Cre*-lineage CINs, in layer 1-2 of the S1 region, with recording solution (32.8±0.1°C) containing (in mM): 125 NaCl, 25 NaHCO_3_, 1.25 NaH_2_PO_4_, 2.5 KCl, 1 MgCl_2_.6H_2_O, 2.5 CaCl_2_-2H_2_O and 10 glucose. Recording electrodes were pulled (Narishige, PC-100) from fabricated standard-wall borosilicate glass capillary tubing (G150F-4, Warner Instruments; OD: 1.50 mm; ID: 0.86 mm) and had 4.3±0.1 MΩ tip resistance when filled with an intracellular solution containing (in mM): 140 potassium gluconate, 10 KCl, 1 MgCl_2_, 10 HEPES, 0.02 EGTA, 3 Mg-ATP and 0.5 sodium-GTP. The pH was adjusted to 7.35 with KOH and osmolarity to 290-300 mOsmol/l with sucrose. Neurons with an access resistance of 10-25 MΩ were considered for recording and the access resistance was monitored, and recordings with >20% change were excluded from subsequent analysis. Signals were acquired at 10 KHz with a low-noise data acquisition system (Digidata 1550B) and a Multiclamp700-A amplifier and were analyzed using pClamp11.1 (Molecular Devices).

### GAD67 immunofluorescence intensity measurements

E15.5 coronal tissue sections from WT and *bRaf^ca^* genotypes were labeled for GAD67. Using Fiji software, 150×150 pixel square boxes were drawn over dorsal, ventral or lateral ganglionic eminence regions and mean fluorescence intensity recorded. The intensity of either the dorsal or ventral regions were divided by the lateral ganglionic eminence area of the same tissue to determine changes in fluorescence intensity.

### Immunofluorescence staining

Adult mice were transcardially perfused with PBS, followed by 4% paraformaldehyde (PFA). The brains were removed and postfixed in PFA for 30 min. Embryonic brains were fixed in 4% PFA for 1 h. Brains were transferred to 30% sucrose for cryoprotection after fixation, embedded in optimal cutting temperature compound and then coronally sectioned using a Tissue-Tek Cryo3 cryostat; adult brains were sectioned at 25 µm and embryonic/early postnatal at 20 µm. Sections were permeabilized in a wash of PBS with 0.3% Triton X-100, then blocked with the same solution containing 5% bovine serum albumin. Primary antibodies were either applied for 1 h at room temperature or overnight at 4°C, followed by three washes in PBS with 0.3% Triton X-100. Secondary antibodies were applied for 1-2 h at room temperature, followed by three washes in PBS with 0.3% Triton X-100 and mounting with VECTASHIELD (Vector Laboratories). Primary antibodies were: sheep anti-ARX (R&D Systems, AF7068, 1:500), mouse anti-GAD67 (MilliporeSigma, MAB5406, 1:500), mouse anti-LHX6 (Santa Cruz Biotechnology, sc-271433, 1:200), rabbit anti-PV (Swant, PV27, 1:400), mouse anti-SATB1 (Santa Cruz Biotechnology, sc-376096, 1:500), rat anti-SST (MilliporeSigma, MAB354, 1:200), rabbit anti-SST (Thermo Fisher Scientific, PA5-85759, 1:500; only used at P2). Secondary antibodies (used at 1:300) were either Alexa 488 or 647 conjugated and from Thermo Fisher Scientific (donkey anti-rabbit 488, A32790; donkey anti-mouse 488, A21202; donkey anti-rat 488, A21208; donkey anti-sheep 488, A11015; goat anti-rabbit 647, A21244; donkey anti-mouse 647, A31571). All antibodies were validated by the company or in-house by proper size on western blot, immunofluorescence signal (loss in knockout) or by expression in cell lines. DAPI-stained nuclei were visualized with NucBlue™ (Thermo Fisher Scientific, R37606). Analyses were confined to neocortical S1 [*y* (−2.0), *x* (3.0), *z* (1.8-1.2)] coordinates for cell counts, except for MGE transplants, where all of the neocortex was assessed.

### Imaging

Fluorescence images were acquired using a Leica DM2000 microscope with mounted DFC3000G camera. Primary culture images were acquired using a Zeiss 800 laser scanning confocal microscope. Fluorescence images were adjusted for brightness/contrast and merged using Fiji software.

### MGE cell transplants

E13.5 MGE tissue was harvested and dissociated to single-cell suspension and then centrifuged at ∼700 ***g*** for 3 min to concentrate the cells. Next, most supernatant liquid was removed and the cells were front-loaded into glass capillaries with 45° beveled tips, as previously described (Vogt et al., 2015a). Neonatal pups were anesthetized on ice and then the loaded capillary punctured the dorsal aspect of the pup's head to access the neocortex, ∼100 µm below the dorsal surface. Cells were then infused into the neocortex and this procedure was repeated at two or three other neocortical sites. Sites were roughly 1 mm apart and formed a line 1 mm from the midline in the right hemisphere. Because these cells migrate extensively in the neocortex (Alvarez-Dolado et al., 2006), these regions were targeted to assure roughly equal separation of boluses. The pup was then warmed and put back with the litter; the transplanted cells developed *in vivo* for 35 days before analysis. Only hosts in which we could assess at least 50 transplanted cells were considered for analysis. MGEs from a single embryo were transplanted into a single WT pup, with the operator unaware of the treatment groups, and embryonic tissue genotyped later.

### Primary cultures

E13.5 MGE tissue was harvested and cultured as previously described (Wundrach et al., 2020). Briefly, glass coverslips were coated with poly-L-lysine, followed by laminin. MGE tissue was mechanically dissociated by trituration using a P1000 pipette tip and seeded at a density of ∼200,000 cells per cm^2^. Cells were seeded in DMEM with 10% fetal bovine serum and changed to Neurobasal medium containing glucose, glutamax and B27 (Vogt et al., 2015a; Wundrach et al., 2020) the next day. Selumetinib (Selleckchem S1008, 20 µM) was applied with new media every other day, as was vehicle (DMSO). Cells were fixed in 4% PFA on day 8 and subjected to immunofluorescence staining. Antibodies used are listed in the ‘Immunofluorescence staining’ section above.

### Soma size quantification

Transplanted MGE cells that developed for 35 days were imaged for tdTomato fluorescence and then somas were traced using Fiji software and the area calculated. Traces were made from 75 different cells/genotype and represent three independent transplanted brains per genotype.

### Statistics

We assessed three or four animals per assessment, as these group sizes have been sufficient to determine significance in previous studies (Elbert et al., 2019; Vogt et al., 2014; Wundrach et al., 2020). No animals were excluded and both male and female mice were used. In most cases, data points were assessed with the operator unaware of treatment groups. Individual data points are presented for all graphs. Normally distributed data were analyzed by two-tailed *t*-test or one-way ANOVA using GraphPad Prism version 7. Chi-squared analyses were performed for normalized data (proportions).

### Western blots

E15.5 forebrains were dissected/frozen on dry ice and then lysed in standard RIPA buffer with protease and phosphatase inhibitors and combined with Laemmli buffer (Bio-Rad, 1610737EDU) containing 2-mercaptoethanol and incubated at 95°C for 5 min. Equal amounts of protein lysates were separated on 10% SDS-PAGE gels and then transferred to nitrocellulose membranes. The membranes were washed in Tris-buffered saline with 0.1% Tween 20 (TBST) and blocked for 1 h in TBST containing 5% non-fat dry milk (blotto, sc-2324 Santa Cruz Biotechnology). Membranes were incubated with primary antibodies overnight at 4°C, washed three times with TBST, incubated with secondary antibodies for 1 h at room temperature and then washed three more times with TBST. Membranes were incubated in ECL solution (Bio-Rad Clarity substrate, 1705061) for 5 min and chemiluminescent images obtained with a Bio-Rad Chemidoc™ MP imaging system. Antibodies (all used at 1:4000) were: rabbit anti-pCREB^Ser133^ (Cell Signaling Technology, 9198), rabbit anti-DLX2 (gift from John Rubenstein, University of California, San Francisco, USA; Lindtner et al., 2019), rabbit anti-GAD65/67 (Sigma-Aldrich, G5163), rabbit anti-GAPDH (Cell Signaling Technology, 2118), mouse anti-LHX6 (Santa Cruz Biotechnology, sc-271433), rabbit anti-MAFB (Sigma-Aldrich, HPA005653), rabbit anti-NKX2-1 (abcam, ab76013), mouse anti-SATB1 (Santa Cruz Biotechnology, sc-376096), rabbit anti-SOX6 (abcam, ab30455), goat anti-rabbit HRP (Bio-Rad, 170-6515) and goat anti-mouse HRP (Bio-Rad, 170-6516). Uncropped membranes are shown in Fig. S6.

## Supplementary Material

10.1242/develop.201371_sup1 Supplementary information
