# Supplementary material for: Distinct hyperactive RAS/MAPK alleles converge on common GABAergic interneuron core programs
Source: Development. 2023 May 31;150(10):dev201371. doi: 10.1242/dev.201371 (PMC10281549; doi:10.1242/dev.201371)
Supplement: Supplementary information [file develop-150-201371-s1.pdf]

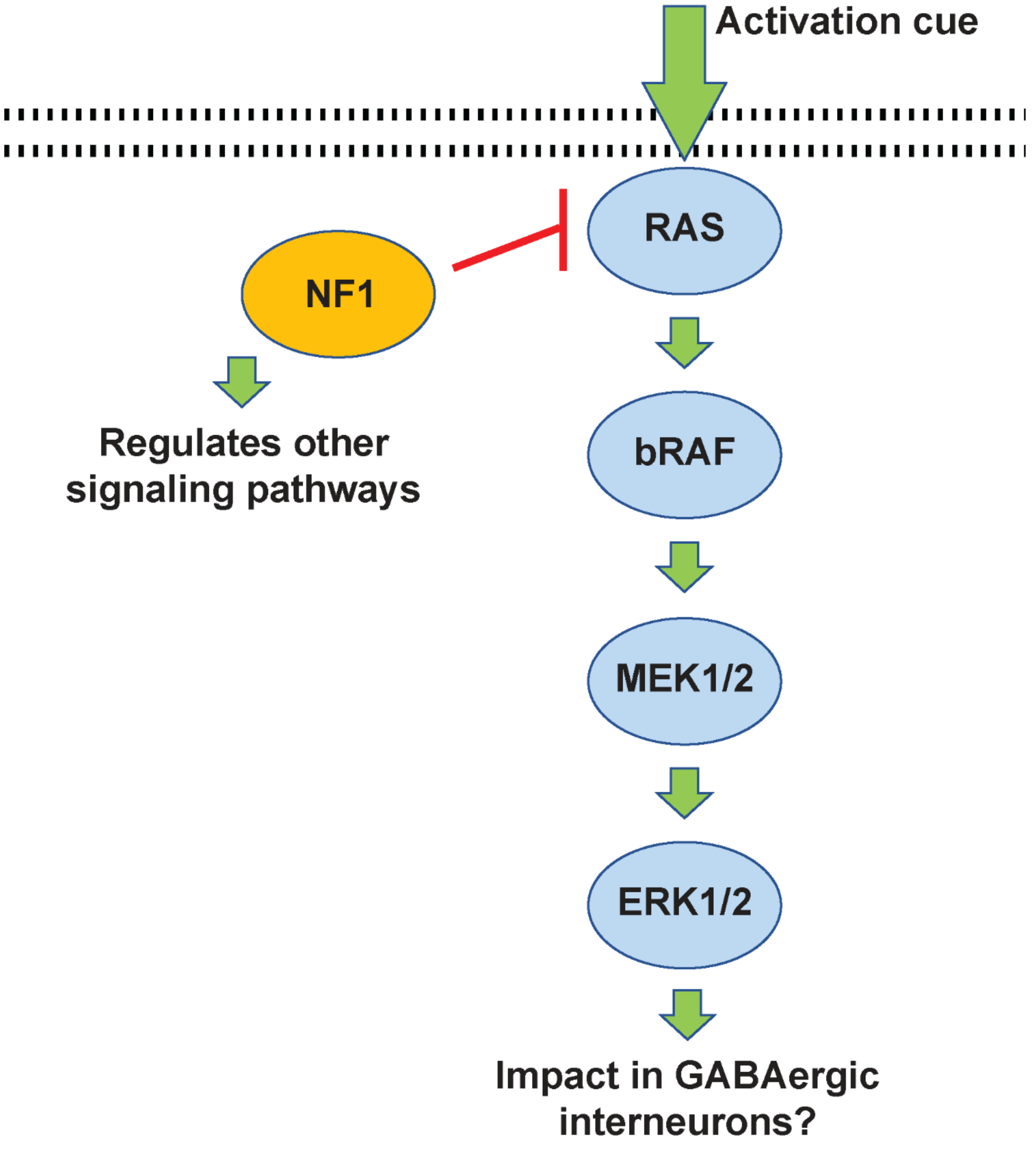

Fig. S1. RAS/MAPK signaling diagram: The RAS/MAPK cascade is induced by extracellular signals, including growth factor recruitment and neuronal activity (activation cue). The RAS protein is the first factor activated that triggers this cascade, with RAF proteins (bRaf shown here) activated by RAS, followed by MEK1/2 and then ERK1/2 in sequence. ERK1/2 activation leads to multiple changes in the cytosol and nucleus but the impact in GABAergic cortical interneurons is not known. Note that this pathway is inhibited/shut down by NF1 activity. Since NF1 also regulates other cellular signaling events, it could influence phenotypes via multiple routes.

Development • Supplementary information

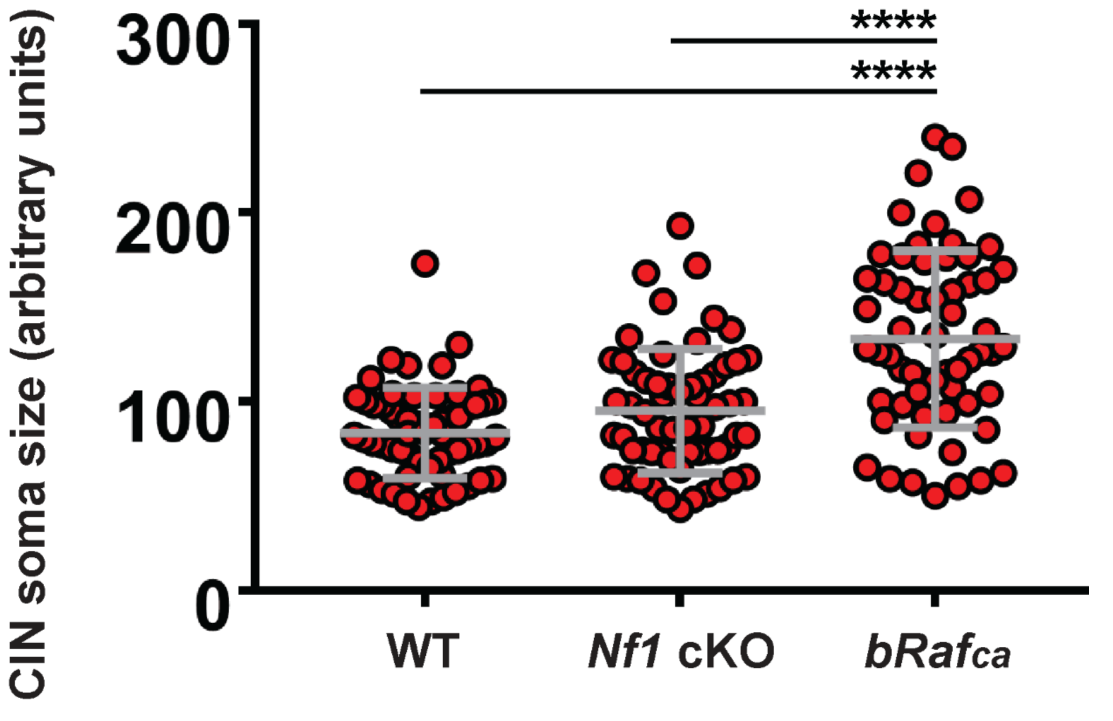

Fig. S2. *bRaf^ca^* soma size is increased in MGE transplanted CINs: E13.5 MGE cells were transplanted in P2 WT neocortices and developed for 35 days. Cells were then assessed for soma area for each genotype. Data are expressed as the mean ± SEM, n = 3 transplants with 75 total cells measured for each genotype. **** p<0.0001.

Development • Supplementary information

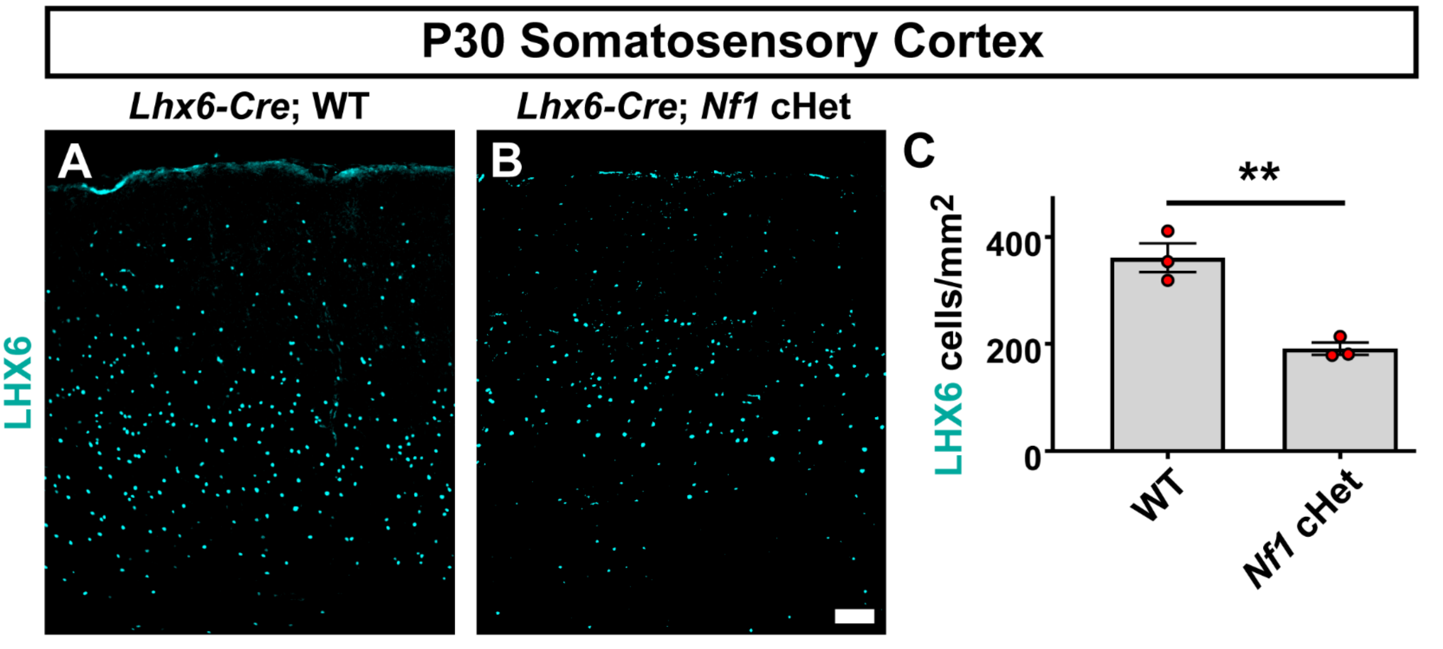

Fig. S3. Reduction of *Nf1* in *Lhx6-Cre* lineage CINs results in reduced LHX6 expression: WT (*Lhx6-Cre* negative) and *Nf1* cHet (*Lhx6-Cre* positive; *Nf1Flox/+*) CINs were assessed at P30 for expression of LHX6 (A, B) in the somatosensory cortex. Quantification of the cell density for LHX6+ revealed a decrease, shown in (C). Data are expressed as the mean ± SEM, n = 3 for each group. ** p<0.01. Scale bar in (B) = 100µm.

Development • Supplementary information

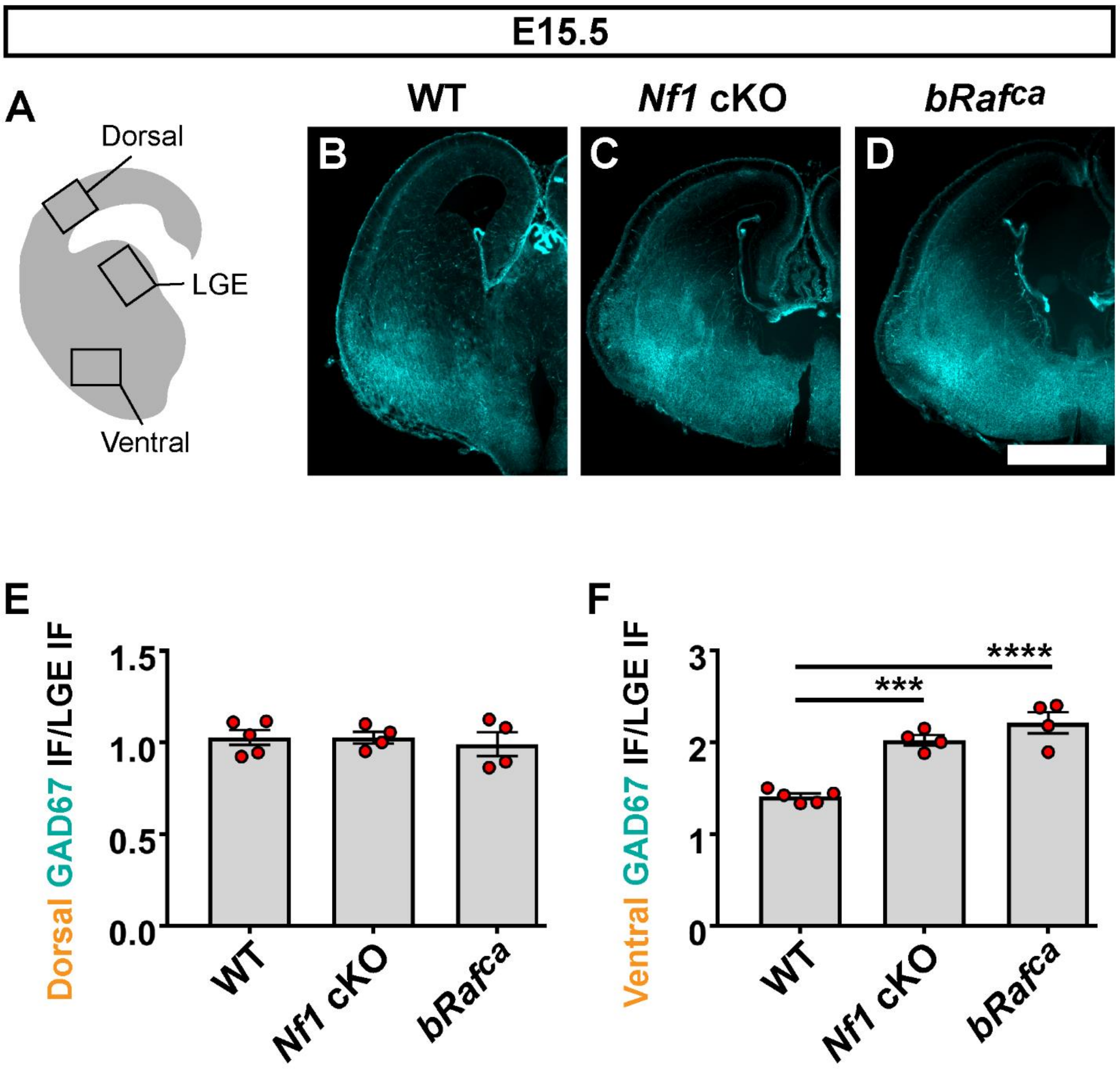

Fig. S4. GAD67 expression is elevated in ventral but not dorsal regions of the brain at E15.5: (A) Schema showing regions of coronal E15.5 sectioned tissue used to assess GAD67 immuno-fluorescent (IF) intensities. Dorsal regions that harbor migrating CINs and ventral regions containing MGE-derived nuclei were measured and normalized to internal LGE progenitor domains that have little to no GAD67 labeling at this age. (B-D) example GAD67 expression in coronal sections of the E15.5 brain for WT, *Nf1* cKO and *bRaf^{ca}*. IF quantification for dorsal and ventral GAD67 expression shows no change in dorsal expression (E) but elevated levels in ventral regions (F). Data are expressed as the mean ± SEM, n = 5 (WT) and n = 4 (*Nf1* cKO and *bRaf^{ca}*), E15.5 brains assessed for each group. *** p<0.001 and **** p<0.0001. Scale bar in (D) = 300µm.

Development • Supplementary information

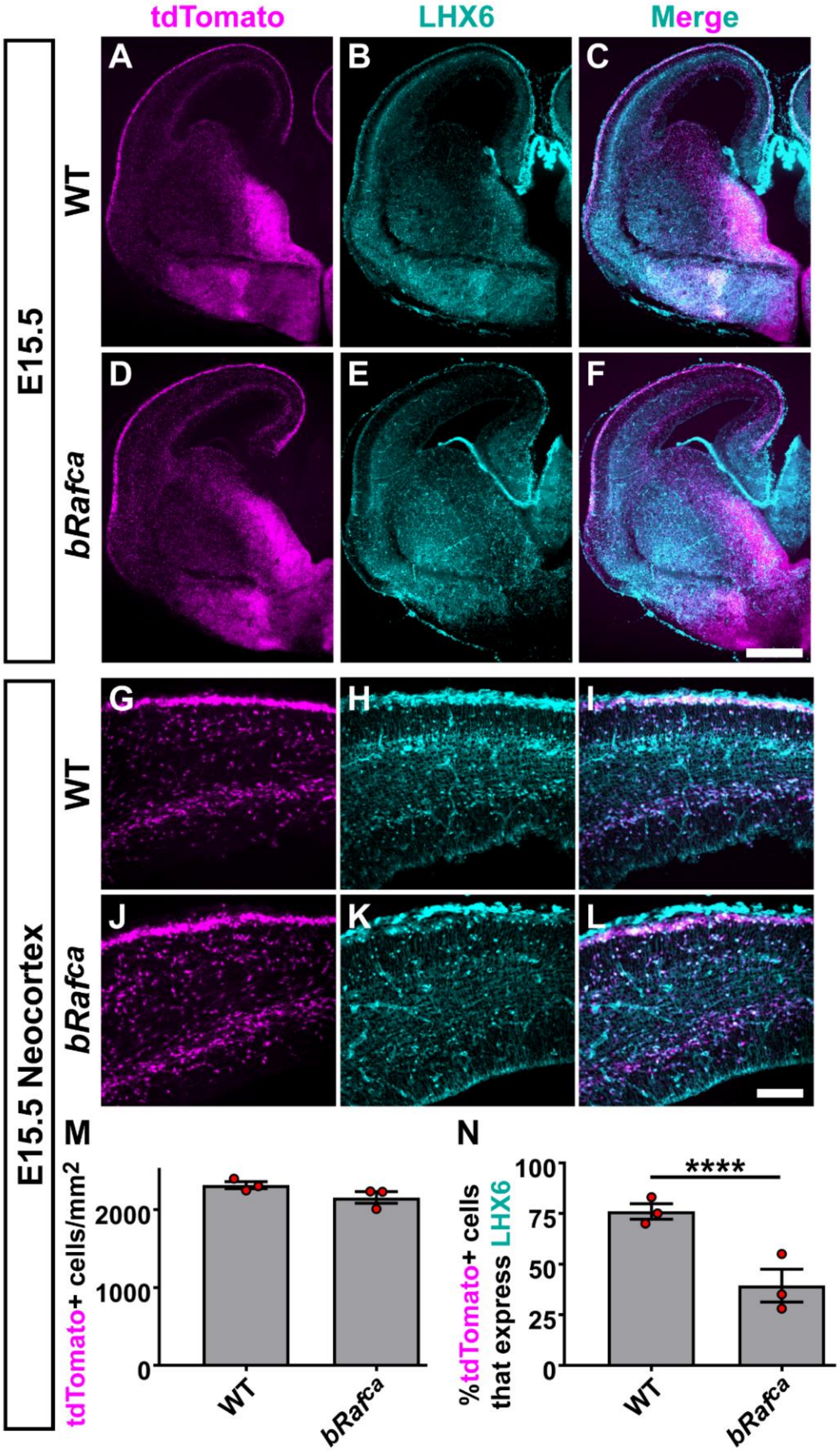

Fig. S5. Reduced LHX6 expression in *Braf*^{ca} neocortices at E15.5: Coronal immuno-fluorescent images labeled for tdTomato and LHX6 from WT (A-C) or *Braf*^{ca} (D-F) embryos. Higher magnification images from the neocortex are shown for WT (G-I) and *Braf*^{ca} (J-L). (M) Quantification of tdTomato cell density from the neocortex reveal no change between genotypes. (N) Quantification of the proportion of tdTomato+ cells that express LHX6 from the neocortex show a decrease in the *Braf*^{ca}. Data are expressed as the mean ± SEM, n = 3 E15.5 brains assessed for each group. **** $p<0.0001$. Scale bars in (F) = 500µm and (L) = 100µm

Development • Supplementary information

Uncropped western blots

DLX2

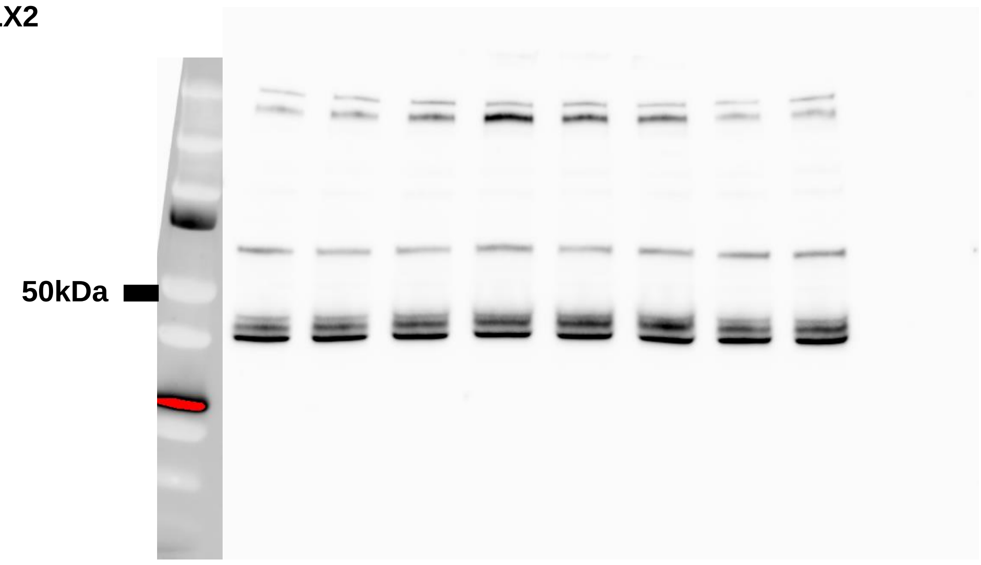

GAD65/65

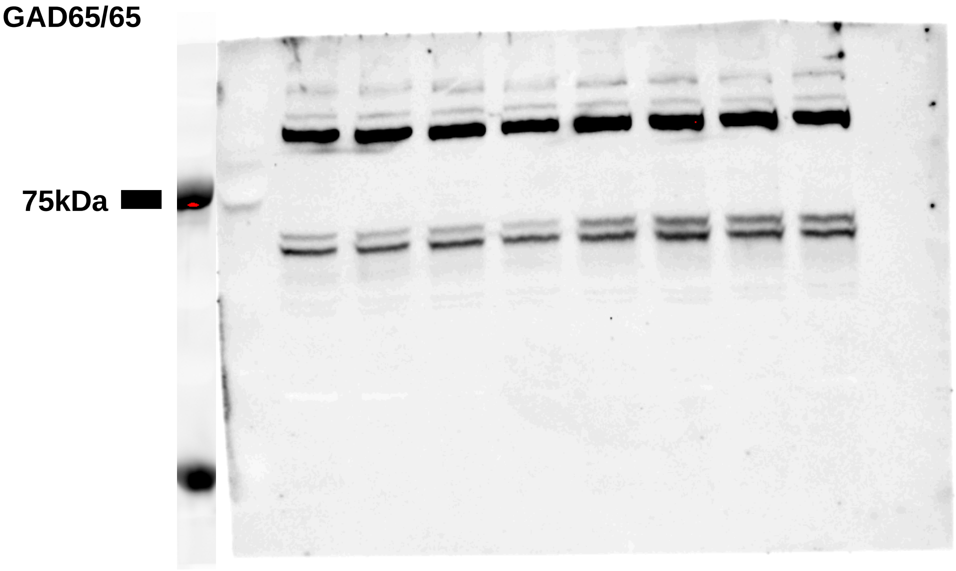

NKX2.1

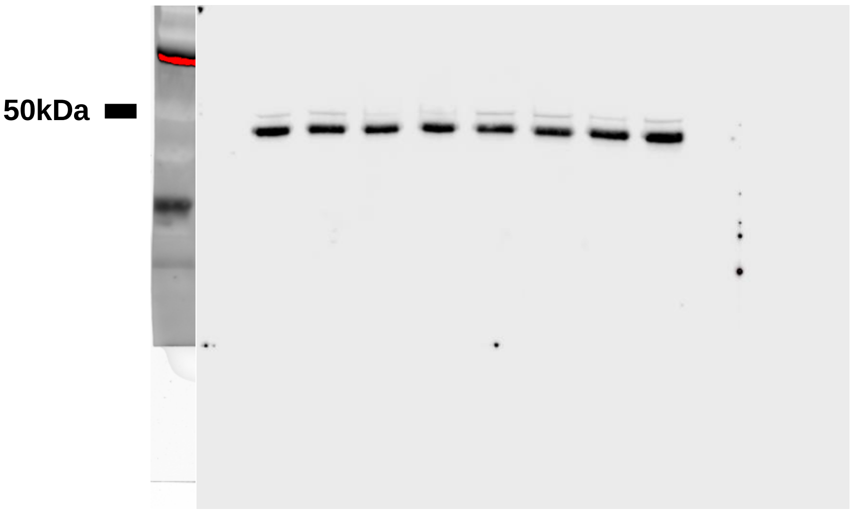

LHX6

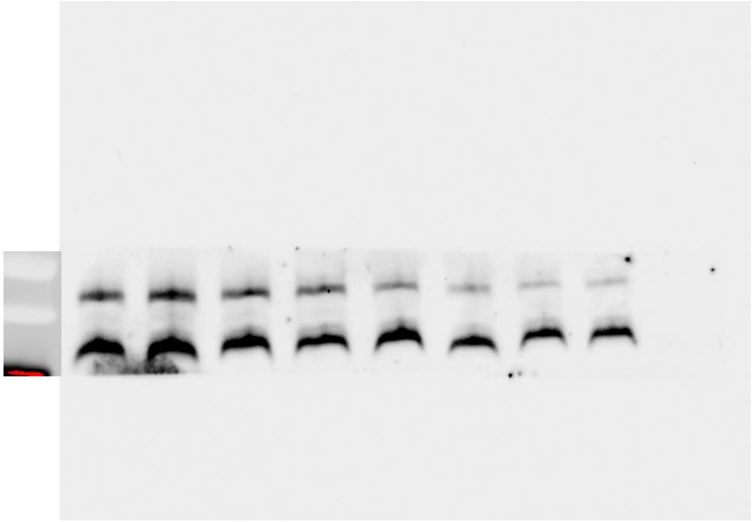

SOX6

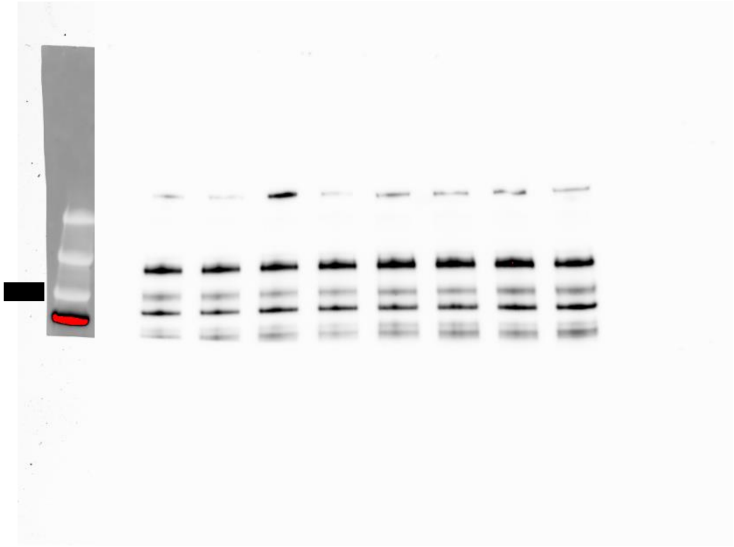

MAFB

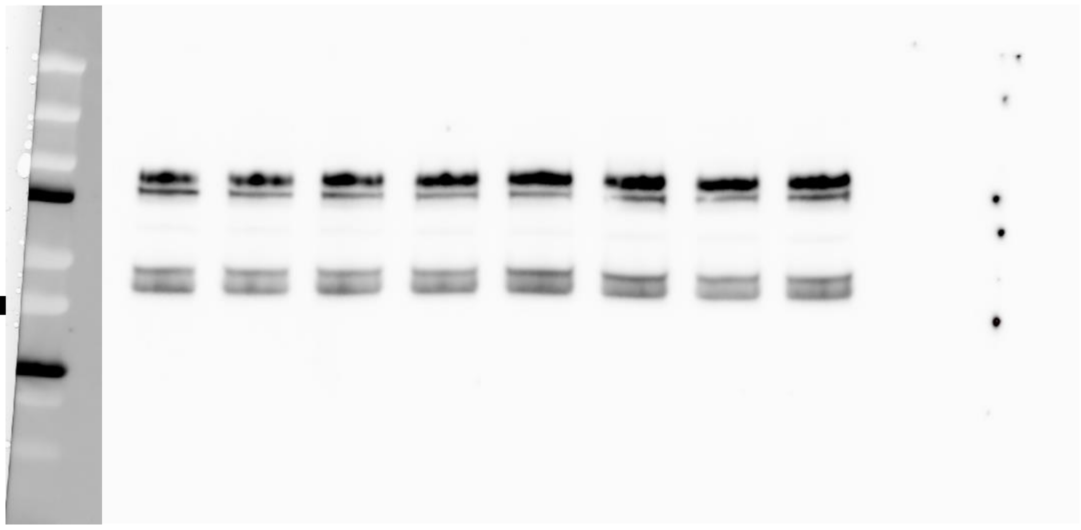

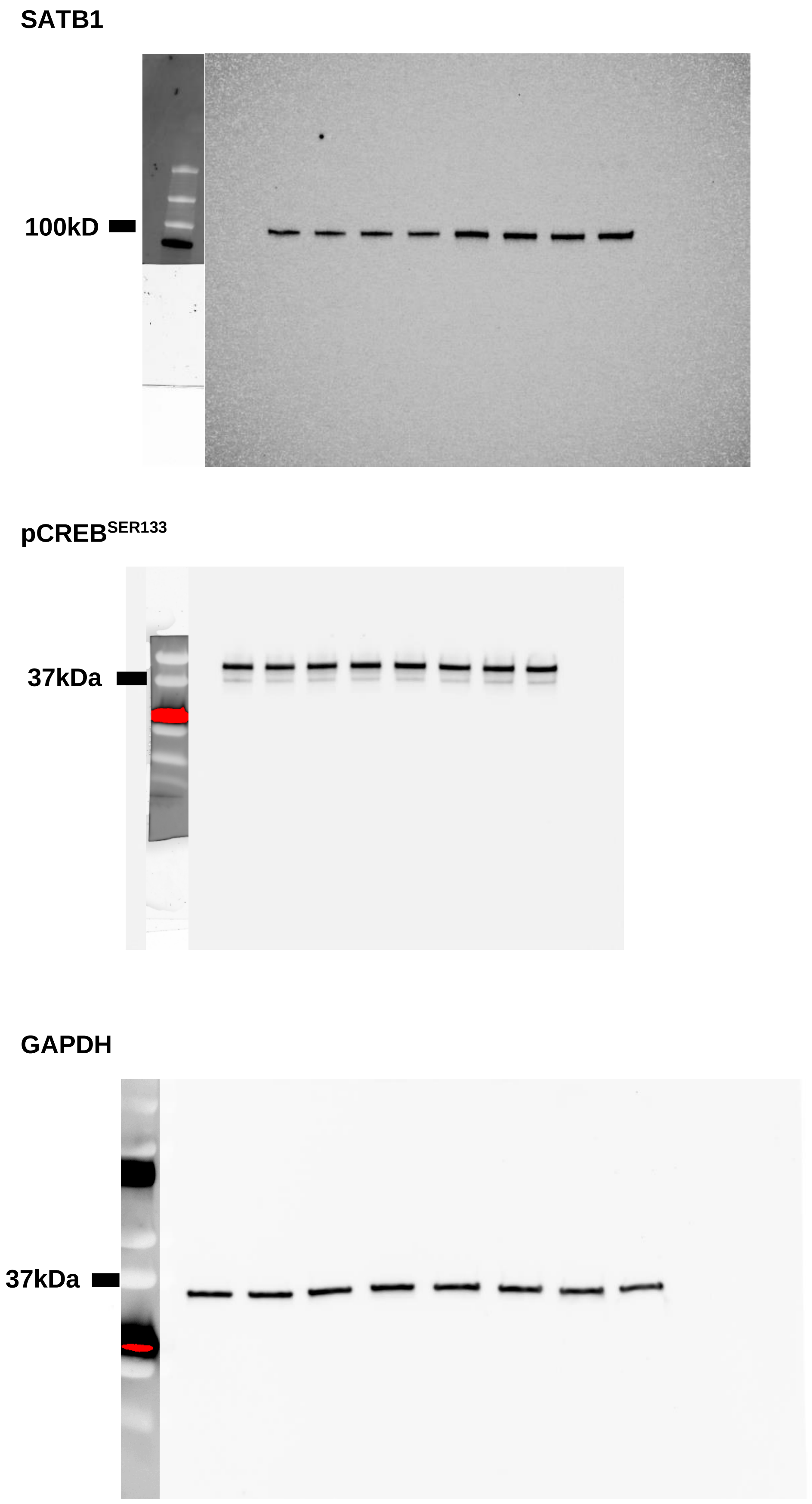

Fig. S6. uncropped western blots.

Table S1. MGE cell transplant count data, related to Figures 1&2: Displayed are the total number of cells counted for the MGE transplant data presented in Figures 1&2. The numbers are the sum of tdTomato+ cells analyzed to determine proportions that expressed either PV, SST or LHX6. LV (lentivirus).

Genotype	Measurement	# of tdTomato+ cells counted
WT *Nkx2.1-Cre*	PV	402
	SST	385
	LHX6	394
Nf1 cKO *Nkx2.1-Cre*	PV	330
	SST	356
	LHX6	287
bRaf^{ca} *Nkx2.1-Cre*	PV	1,149
	SST	1,305
	LHX6	824
WT LV *Dlxi1/2b-Cre*	PV	212
	SST	350
bRaf^{ca} LV *Dlxi1/2b-Cre*	PV	507
	SST	429

Development • Supplementary information

Table S2. Active and passive electrophysiological properties from WT and *bRaf*^{ca} transplanted cells: Chart lists measurements from transplanted WT and *bRaf*^{ca} cells. Abbreviations: AP (action potential), Cm (membrane capacitance), pF (picofarads), Rm (resting membrane capacitance), MΩ (mega ohm), Ra (active membrane capacitance), RMP (resting membrane potential), mV (millivolt), ms (millisecond).

**Measurements (Units)**	**WT**	***bRaf*^{ca}**	**p value**
**Cm (pF)**	**54.9**	**61.1**	
SEM	±2.5	±4	**n.s.**
n	24	31	
**Rm (MΩ)**	**440.6**	**470.5**	
SEM	±33.8	±32.6	**n.s.**
n	24	31	
**Ra (MΩ)**	**16.9**	**18.7**	
SEM	±0.7	±0.6	**n.s. (0.07)**
n	24	21	
**RMP (mV)**	**-61.7**	**-58.8**	
SEM	±1.4	±1.3	**n.s.**
n	24	24	
**Depolarization to 1st AP (mV) at 400pA**	**15**	**21.4**	
SEM	±0.7	±1	****** < 0.0001**
n	24	31	
**Time to 1st AP (ms) at 400pA**	**1.2**	**2.9**	
SEM	±0.1	±0.6	*** 0.01**
n	24	31	
**1st interspike interval (ms)**	**9.1**	**15.9**	
SEM	±0.5	±2.8	*** 0.04**
n	24	31	
**Last interspike interval (ms)**	**30.3**	**42.1**	
SEM	±7.7	±7.8	**n.s.**
n	24	31	
**Spike frequency adaption ratio**	**3.4**	**2.7**	
SEM	±0.9	±0.3	**n.s.**
n	24	31	
