# Supplementary material for: Distinct hyperactive RAS/MAPK alleles converge on common GABAergic interneuron core programs
Source: Development. 2023 May 31;150(10):dev201371. doi: 10.1242/dev.201371 (PMC10281549; doi:10.1242/dev.201371)
Supplement: Supplementary file 1 [file develop-150-201371_review_history.pdf]

Distinct hyperactive RAS/MAPK alleles converge on common GABAergic interneuron core programs

Sara J. Knowles, April M. Stafford, Tariq Zaman, Kartik Angara, Michael R. Williams, Jason M. Newbern and Daniel Vogt

DOI: 10.1242/dev.201371

Editor: Debra Silver

Review timeline

Original submission:	20 October 2022
Editorial decision:	28 November 2022
First revision received:	3 April 2023
Editorial decision:	24 April 2023
Second revision received:	24 April 2023
Accepted:	26 April 2023

Original submission

First decision letter

MS ID#: DEVELOP/2022/201371

MS TITLE: Distinct hyperactive RAS/MAPK alleles converge on common GABAergic interneuron core programs

AUTHORS: Sara J. Knowles, April M. Stafford, Tariq Zaman, Kartik Angara, Michael R. Williams, Jason M. Newbern, and Daniel Vogt

I have now received all the referees' reports on the above manuscript, and have reached a decision. The referees' comments are appended below, or you can access them online: please go to BenchPress and click on the 'Manuscripts with Decisions' queue in the Author Area.

As you will see, the referees express considerable interest in your work and were favorable about the study. However, they also have some significant criticisms and recommend a substantial revision of your manuscript before we can consider publication. You will see the reviewer concerns attached below which make recommendations to bolster the conclusions of your study. In particular, reviewer 1 raises concerns that some findings may be preliminary and suggests several important experiments to address this including the need for more replicates, addressing the nature of recorded neurons, and clarity of analyses and methods. Reviewers 2 and 3 also suggest the need for more experimental detail and clarity of some points, and both ask for further clarity as to whether Ras/MapK signaling is most relevant in progenitors versus neurons.

If you are able to revise the manuscript along the lines suggested, I will be happy receive a revised version of the manuscript. Your revised paper will be re-reviewed by one or more of the original referees, and acceptance of your manuscript will depend on your addressing satisfactorily the reviewers' major concerns. Please also note that Development will normally permit only one round of major revision. If it would be helpful, you are welcome to contact us to discuss your revision in greater detail. Please send us a point-by-point response indicating your plans for addressing the referee’s comments, and we will look over this and provide further guidance.

© 2023. Published by The Company of Biologists under the terms of the Creative Commons Attribution License (https://creativecommons.org/licenses/by/4.0/).

Please attend to all of the reviewers' comments and ensure that you clearly highlight all changes made in the revised manuscript. Please avoid using 'Tracked changes' in Word files as these are lost in PDF conversion. I should be grateful if you would also provide a point-by-point response detailing how you have dealt with the points raised by the reviewers in the 'Response to Reviewers' box. If you do not agree with any of their criticisms or suggestions please explain clearly why this is so.

Reviewer 1

Advance summary and potential significance to field

In this manuscript, Knowles et al. investigate the role of RAS/MAPK signaling in the regulation of cortical interneuron differentiation. Using mouse genetics embryonic transplantation and pharmacology, the authors show that two different manipulations of this pathway (Nf1 loss of function and bRaf constitutive activation) lead to a developmental imbalance in interneuron fates with a decrease in the proportion of cortical interneurons that express LHX6 and parvalbumin, and a selective increase in the density of somatostatin and SATB1-expressing interneurons (albeit the former phenotype is transient in Nf1cKO mice). Using two different mouse lines to time the ablation of these genes (Nkx1Cre and Lhx6Cre), the authors showed that the difference in gene expression levels is not due to an impairment in progenitor proliferation. Finally increased SST expression in the RAS/MAPK mutants can be rescued by treatment with MEK blockers. In in vitro experiments, Selumetinib treatment normalizes Sst expression after neuronal depolarization. Although these results are interesting and suggest that the RAS/MAPK cascade may mediate some aspects of activity-dependent interneuron differentiation, there are several caveats that need to be addressed.

Comments for the author

Major points:
-The study is underpowered with several experiments consisting of only 3 replicas. It is unclear why the P2 and P7 SST cIN density analyses in Fig3M-R show minimal standard deviation. Variability in interneuron density among different brains is expected since these are developmental stages in which interneuron migration is still occurring. What is the variability in the density of transplanted interneurons among control and mutant brains?

-The experimental strategy for stereology is not clearly stated in the paper.
The authors indicate that neocortical tissue was analyzed but interneuronal density varies by cortical area, laminar position and developmental age. In addition, stereotactic bregma coordinates should be provided.

-The methodology for the transplant experiments is not described in the paper.
The location of the injections as well as the efficiency of the transplantation should be included.

-The effectiveness of the KCl treatment to upregulate FOSB expression (Figure S6) appears very limited with only activation in 10 % of the cells in culture.
The authors should discuss this limitation and consider using an alternative approach to manipulate activity and bolster their argument.

-In Figure 2, it is unclear how the recorded interneurons were identified. What is the laminar location of these neurons? Were recordings limited to one lamina?
Furthermore, the posthoc biochemical and morphological identity of these neurons should be added to the manuscript. Given the subtype diversity of Sst and PV interneurons, without this distinction differences attributed to genotype may reflect subtype differences in electrophysiological properties.

-Fig. S3 shows a reduction of caspase 3- positive interneurons in bRafca mutants, supporting the interpretation of increased Sst cIN survival. However the mechanism underlying this process is unclear. Are cell survival and activity-dependent SST expression regulated by different signaling cascades?

© 2023. Published by The Company of Biologists under the terms of the Creative Commons Attribution License (https://creativecommons.org/licenses/by/4.0/).

- Fig. 5G shows a very significant increase in SATB1 expression in the Ras/MAPK mutants but this difference is not apparent in the representative sections shown in 5F compared to 5B.

Minor points:
-In Fig.3M, the authors report an increase in Sst cIN density in Nf1 mutants at P2-7. Could increase tangential or radial migration contribute to this phenotype?
-The authors previous work (Vogt et al 2015) showed that Pten deletion in cIN leads to a reduction of SST cINs. Could an antagonistic regulation between PTEN and bRaf explain this phenotype?

-Previous research suggest that (Petros et al Cell Reports 2015) interneuron progenitors are biased to PV or Sst interneuron fates according to their apical vs basal position in the ganglionic eminence. This is in contrast with the present findings indicating that RAS/MAPK signaling could bias interneuron subtype identity in postmitotic neurons. The authors should discuss this.

Reviewer 2

Advance summary and potential significance to field

The manuscript submitted by Knowles et al., uses an elegant comparative genetic approach combined with transplantation and in vitro culture system to understand the impact of increased RAS/MAPK signaling on cortical interneuron development. The authors target Nf1 loss (upstream of RAS) and a constitutively active bRaf (downstream of RAS) during cortical interneuron development using both Nkx2.1-cre (earliest MGE progenitors) and Lhx6-cre (postmitotic neurons). In summary, their findings reveal that cortical interneuron development is altered after genetic activation of the RAS/MAPK pathway. MGE progenitor cells targeted for Nf1 loss and bRaf-CA have reduced Lhx6, ARX, and PV expression with subsequent increased expression of SST+, Satb1+ and GAD65/67. Also, lowering the MAPK pathway with Selumetinib restored SST+ expression to control levels in MGE primary cultures. The authors show that neural activity is also impacted in the bRAF-CA experiments with lower action potential spiking frequencies. In addition, MGE cultures treated with KCL to increase neural activity show induction of SST expression. However, treatment of Selumetinib and KCL did not result in induction of SST expression suggesting a critical role in the MAPK pathway.

This manuscript reveals new information in the field of cortical interneuron development and RAS/MAPK signaling. The findings show common phenotypes caused by increased RAS/MAPK signaling during cortical interneuron development and reveal mechanisms that link the impact of abnormal MAPK signaling with transcription factor expression in cINs.

The data are important for two key reasons:

1. -revealing new basic mechanisms key to understanding cortical interneuron development.

2. -describing novel phenotypes observed after RAS/MAPK pathway activation which is relevant to RASopathy syndromes that exhibit neurocognitive abnormalities.

The experiments are clearly described, and the data are of high quality. However, I have a few points the authors should address or consider in the manuscript. I also have a couple minor points for the authors to address.

Comments for the author

Major Points:

-The authors show that Gad65/67 levels are increased in the E15.5 Nf1 loss or bRaf-ca- using western blot analysis. However, the dissection was the entire forebrain including cortex and striatum. Can the authors show or comment if the distribution of Gad65 or Gad67 cells are changed in the cortex after Nf1 loss or bRaf-ca at embryonic stages. Is the increase in expression from increased positive cells in the ventral or dorsal telencephalon?

© 2023. Published by The Company of Biologists under the terms of the Creative Commons Attribution License (https://creativecommons.org/licenses/by/4.0/).

-Related to the previous point, the authors show the reduction in Lhx6 expression but normal tdTomato expression at E15.5 in bRaf-ca embryos. The images in Supp. Fig4 (J compared to G) look to have increase tdTomato expression in the outer migratory pathway. It might be informative to examine the outer and inner migratory pathways of cINs. In fact, the outer cells appear to be the more sensitive region for the increased Satb1 cells in Figure 6. Can the authors comment if the Satb1 (and dtTomato fate map) have regional difference in the migratory pathway going to the cortex?

-The authors should consider some discussion about the models used in this study. For example, the bRAF activation model relies on the V600E mutation observed in cancers and the NF1 results are largely presented from biallelic deletion and not heterozygous. Do the authors think there is a necessary threshold of MAPK pathway activation for distinct phenotypes? What about other models of RAS/MAPK activation that model RASopathy syndromes. Potentially this point could be expanded in the discussion.

-The authors make the following point in the last paragraph of the discussion:

"This was not observed in the neocortex of bRaf hyperactive mutants (Data not shown) and could indicate other Nf1-regulated events 7 may influence ventral-derived oligodendrocytes"

However, the authors do not show any data on the oligodendrocyte analysis including what markers were used and what stages were analyzed. If the authors want to include this statement, then this data would need to be included in the results section or supp Data section. Alternatively, the authors could expand the discussion to compare recent RASopathy/MAPK activation models and the effect on oligodendrocytes vs cINs (or other cells).

-the authors use both Nkx2.1-cre (Fig. 1) and Lhx6-cre (supp fig. 2) to parse progenitor vs postmitotic targeting of NF1 loss or Braf-ca in the manuscript. The authors should clearly describe which cre is used for Figure 3. One option is to include a sentence after the Lhx6-cre description for the supp. data that all remaining figures are on the Nkx2.1-cre driven deletion/activation. Also, do the Lhx6-cre Nf1 cKOs survive to E18? Can the authors comment in more detail on how the phenotype is different from the Nkx2.1-cre experiments? This is an important point given the difference in timing for cre expression (Nkx2.1-cre early and Lhx6-cre in post-mitotic cINs).

Minor Points:

-could the authors comment if the morphology is changed in the in vitro SST+ cells in Braf-ca compared to NF1 loss or control in Figure 3B-D.

-Have the authors examined MAPK pathway activation in the MGE cultures or transplant experiments. Do the increased SST+ cells express phosphor-ERK1/2 or MAPK target genes?

-there are several Supp. Data figures. The authors could consider adding the cell death data (Supp figure 3) or ARX data (Supp figure 5) to the main manuscript.

Reviewer 3

Advance summary and potential significance to field

This manuscript examines the role of MapK signaling on the maturation of GABAergic cortical interneurons (CINs). The authors utilize two genetic models that result in increased RAS/MapK signaling including Nf1 loss of function (LOF) and bRaf constitutively active (bRaf-ca) mutant mice to examine postnatal maturation of CINs. Since these mice either don't survive well postnatally (bRaf-ca) or show defects also in non-CINs (e.g. oligos in Nf1 LOF), they make use of a clever transplantation paradigm implanting embryonic MGE progenitors into the postnatal cortex and allow the mutant CINs mature. MGE cells from both genetic models show a reduction in the parvalbumin CINs and a concomitant increase in those expressing Somatostatin (SST) which also exhibit SST-like physiological characteristics, at least at early postnatal time points. Interestingly, they found that bRaf-ca mutant CINs elude cell death resulting in the maintained increases in SST+

© 2023. Published by The Company of Biologists under the terms of the Creative Commons Attribution License (https://creativecommons.org/licenses/by/4.0/).

CINs in the more mature brains. Western blots of embryonic forebrain show increased Gad65/67 levels indicating enhanced GABAergic differentiation. While Dlx proteins are known to specify the GABAergic fate of CINs by directly binding the Gad genes the authors observed no change in their expression, supporting an independent role for RAS/MapK signaling in GABAergic maturation of CINs. Finally, the authors provide data that support neuronal activity-driven MapK signaling leading to the increased GABAergic maturation and also SST expression (via Satb1). Taken together, these findings have relevance for the cognitive deficits observed in patients suffering from RASopathies.

Comments for the author

This is a well-done study that provides important information for the RAS/MapK and forebrain development fields and thus appears appropriate for publication in Development, however, there are few issues that the authors need to address first.

While the authors cite their previous papers using a similar transplantation approach, this manuscript would benefit from more details being provided as to the experiments both in the results and methods sections. For example, I assume that MGEs from the same embryo are dissociated and transplanted into separate animals (postnatal day?) with the genotyping being carried out after the fact.

The question of, at what point in the MGE CIN lineage these changes in RAS/MapK signaling impact CIN subtype maturation, is interesting but not well addressed.
Why wasn't the transplantation technique used for Lhx6-cre experiments, given the observed lethality? Is the Nf1 het an appropriate way to asses this? Ultimately it would be more informative to be able to assess PV/SST proportions in mutant CINs after Lhx6-cre (i.e. postmitotic) recombination. As it stands, the question of whether Nf1 LOF or bRaf-ca in dividing MGE progenitors vs postmitotic CINs contributes to the observed changes in mutant CINs, remains unresolved.

The Satb1 data in Figure 5 would benefit from a more nuanced analysis. While it looks like a similar density of Satb1+ cells occupy the most superficial portion of the cortical plate in both mutants, the intermediate zone shows differential numbers – with more in the bRaf-ca mutants. If this difference is real, it may hint towards the distinct phenotypes seen in the two genetic models.

In my opinion, the Arx data should be included in the main figures as they show a strong and shared phenotype in mutant CINs between the 2 models.

The SST data in vitro are pretty convincing, however, no assessment of PV+ CINs is performed. Can you detect PV+ CINs in vitro and if so, is their phenotype (or ratios) impacted when MapK signaling/neuronal activity is altered?

First revision

<u>Author response to reviewers' comments</u>

Reviewer 1 Advance summary and potential significance to field...
In this manuscript, Knowles et al. investigate the role of RAS/MAPK signaling in the regulation of cortical interneuron differentiation. Using mouse genetics, embryonic transplantation and pharmacology, the authors show that two different manipulations of this pathway (Nf1 loss of function and bRaf constitutive activation) lead to a developmental imbalance in interneuron fates with a decrease in the proportion of cortical interneurons that express LHX6 and parvalbumin, and a selective increase in the density of somatostatin and SATB1-expressing interneurons (albeit the former phenotype is transient in Nf1cKO mice). Using two different mouse lines to time the ablation of these genes (Nkx1Cre and Lhx6Cre), the authors showed that the difference in gene expression levels is not due to an impairment in progenitor proliferation. Finally, increased SST expression in the RAS/MAPK mutants can be rescued by treatment with MEK blockers. In in vitro experiments, Selumetinib treatment normalizes Sst expression after neuronal depolarization.

© 2023. Published by The Company of Biologists under the terms of the Creative Commons Attribution License (https://creativecommons.org/licenses/by/4.0/).

Although these results are interesting and suggest that the RAS/MAPK cascade may mediate some aspects of activity-dependent interneuron differentiation, there are several caveats that need to be addressed.

Reviewer 1 Comments for the author...

Major points:
1) -The study is underpowered with several experiments consisting of only 3 replicas. It is unclear why the P2 and P7 SST cIN density analyses in Fig3M-R show minimal standard deviation. Variability in interneuron density among different brains is expected since these are developmental stages in which interneuron migration is still occurring. What is the variability in the density of transplanted interneurons among control and mutant brains?

Response: The primary histological data herein are not underpowered and an n of 3 is typical when assessing cell counts in the brains of mice on a mostly congenic background, which leads to highly consistent and stereotyped cell migration as well as other milestones.

We do agree there are some data points that could benefit from additional samples and have included more n's for the electrophysiology data (see improved Fig. 3 and Supp. Table 2), where variability could be an issue due to the transplanted cells and ability to acquire sufficient numbers for statistics.

Regarding the P2 and P7 counts, while interneurons are migrating/laminating throughout these ages, these events are highly stereotyped and reproducible, as noted above due to the mice being congenic. Thus, the small variability observed is normal. Due to other suggestions from reviewers, we have now parceled these counts at E15.5 into smaller regions, see edited Fig. 6.

We have also included more data on the cell transplants, which we hope will help readers understand the cell numbers assessed that include the number counted for each group over the 3 transplants done. Unfortunately, cell density is not a reliable measurement for the transplants as we need to sample the entire neocortex, cell density varies depending on proximity to the injection site and cells can be lost during the procedure due to leaking out of the brain during the procedure. However, we hope reporting these numbers will help readers have confidence we did a thorough job, as these numbers are in the hundreds to thousands of cells evaluated. We have included the raw cell counts as new Supp. Table 1.

2) -The experimental strategy for stereology is not clearly stated in the paper. The authors indicate that neocortical tissue was analyzed but interneuronal density varies by cortical area, laminar position and developmental age. In addition, stereotactic bregma coordinates should be provided.

Response: Thank you, we have added more detail about where we imaged, i.e. Somatosensory Cortex (S1) in the non-transplant brains, as well as provided the coordinates for this region in the methods section. Specifically, in non-transplanted animals, we targeted S1 y (-2.0) x (3.0) z (1.8-1.2) coordinates for cellular counts in adult brains; the same developing region was targeted at earlier ages to be consistent.

3) -The methodology for the transplant experiments is not described in the paper. The location of the injections as well as the efficiency of the transplantation should be included.

Response: Thank you for noticing this. We apologize, this was an oversight and we have now added a detailed description about the transplant procedure in the methods. We have included details about neocortical locations. However, since these cells extensively migrate and we are looking at cell autonomous impacts after transplantation, the injection site of the cells in the neocortex does not impact our interpretations. In comment 1, we included data about cell numbers assessed in the transplants to address the efficiency of the procedure. This is a procedure where cells can be lost (mainly due to them leaking out of the brain) so final numbers of cells assessed vary a great deal. Cell counts per sample to compare relative cells between genotypes are reported in new Supplemental Table 1. Our threshold is the assessment of at least 50 cells per transplant host to qualify for data analysis; this threshold measurement is now included in the methods section.

© 2023. Published by The Company of Biologists under the terms of the Creative Commons Attribution License (https://creativecommons.org/licenses/by/4.0/).

4) -The effectiveness of the KCl treatment to upregulate FOSB expression (Figure S6) appears very limited with only activation in 10% of the cells in culture. The authors should discuss this limitation and consider using an alternative approach to manipulate activity and bolster their argument.

Response: Yes, we agree. We think these analyses need further work and are not yet ready for publication and have decided to remove this datum and related activity-dependent data as they are not part of the core story. This is also important to remove as more data are being added to solidify the core story and utilize the space efficiently.

5) -In Figure 2, it is unclear how the recorded interneurons were identified. What is the laminar location of these neurons? Were recordings limited to one lamina? Furthermore, the posthoc biochemical and morphological identity of these neurons should be added to the manuscript. Given the subtype diversity of Sst and PV interneurons, without this distinction differences attributed to genotype may reflect subtype differences in electrophysiological properties.

Response: Our analyses focused on layers 2/3 for consistency; this information has now been added to the methods. Since we are dealing with a cell signaling pathway that alters cell fate/property ratios, we can't reliably do a faithful breakdown of subtypes but rather focused on how each group of transplanted interneurons behaved. Unfortunately, morphology is incredibly unreliable as a determinate of cell fate in WT interneurons, and more so with mutant interneurons when dealing with cell morphology altering pathways. We have added text in the results to bring this point up.

6) -Fig. S3 shows a reduction of caspase 3- positive interneurons in bRafca mutants, supporting the interpretation of increased Sst cIN survival. However, the mechanism underlying this process is unclear. Are cell survival and activity-dependent SST expression regulated by different signaling cascades?

Response: This is a great question. It is beyond the scope of this paper to investigate potential mechanisms linking these two ideas. Our goal in later figures of the paper was to show that RAS/MAPK activity is involved in SST expression in CINs, which the data support. In addition, there is a long line of literature about RAS/MAPK activity and cell survival in the cancer field, which prompted the question about cell death during a known window of apoptosis in these mutant cells. We have added the following reference about RAS/MAPK and cell death: PMID: 10558990.

7) - Fig. 5G shows a very significant increase in SATB1 expression in the Ras/MAPK mutants but this difference is not apparent in the representative sections shown in 5F compared to 5B.

Response: We agree these images can be confusing but in SATB1 labeled panels (B-G), there is an obvious increase in SATB1 expressing cells that correspond to the graphs. The main differences are in the upper stream of migrating cells. There are some auto fluorescent regions in the middle regions of the tissue that add to the confusion but the SATB1+ cells are different between the genotypes. We considered more arrows to highlight the positive cells but chose not to so more of the image was accessible to readers.

Minor points:
8) -In Fig.3M, the authors report an increase in Sst cIN density in Nf1 mutants at P2-7. Could increase tangential or radial migration contribute to this phenotype?

Response: Thank you for this question. We have looked at total cell density of MGE derived cells (using the Cre reporter Ai14/tdTomato) and have not found any changes in tangential migration or total cells in distinct parts of the neocortex (total tdTomato cell density at P2 and P7, also at E15.5). Together, we believe that the change in SST expression observed is due to changes within the interneurons and not by aberrant tangential or radial migration.

9) -The authors previous work (Vogt et al 2015) showed that Pten deletion in cIN leads to a reduction of SST cINs. Could an antagonistic regulation between PTEN and bRaf explain this phenotype?

© 2023. Published by The Company of Biologists under the terms of the Creative Commons Attribution License (https://creativecommons.org/licenses/by/4.0/).

Response: Good question! We can't say no to this idea but we have tried many times to look into crosstalk between these key pathways and have never seen one pathway grossly impacting the other in interneurons or their progenitors (unfortunately, none of it is published because it is all negative data). This is in contrast to many reports using immortalized cancer cell lines that have shown this. Since these immortalized cells have been manipulated in a way that allows them to constantly grow/divide, unlike the interneurons.

However, it is possible that they could lead to antagonistic phenotypes using distinct mechanisms. We have added text to the discussion to suggest that apoptosis seems to be a common antagonistic phenotype that influences SST neurons impacted by both the RAS/MAPK and mTOR pathways.

10) -Previous research suggest that (Petros et al Cell Reports 2015) interneuron progenitors are biased to PV or Sst interneuron fates according to their apical vs basal position in the ganglionic eminence. This is in contrast with the present findings indicating that RAS/MAPK signaling could bias interneuron subtype identity in postmitotic neurons. The authors should discuss this.

Response: This is a good point and has been added to the discussion. These 2 ideas are not mutually exclusive, and several different mechanisms likely contribute to differentiation of SST lineages and their properties. The big take away here is that the Petros et al paper and our data herein do not result in a 100% change in a specific interneuron population, suggesting each of these important findings are contributors to the whole phenotype rather than a singular determinate.

Reviewer 2 Advance summary and potential significance to field...
The manuscript submitted by Knowles et al., uses an elegant comparative genetic approach combined with transplantation and in vitro culture system to understand the impact of increased RAS/MAPK signaling on cortical interneuron development. The authors target Nf1 loss (upstream of RAS) and a constitutively active bRaf (downstream of RAS) during cortical interneuron development using both Nkx2.1-cre (earliest MGE progenitors) and Lhx6-cre (postmitotic neurons). In summary, their findings reveal that cortical interneuron development is altered after genetic activation of the RAS/MAPK pathway. MGE progenitor cells targeted for Nf1 loss and bRaf-CA have reduced Lhx6, ARX, and PV expression with subsequent increased expression of SST+, Satb1+ and GAD65/67. Also, lowering the MAPK pathway with Selumetinib restored SST+ expression to control levels in MGE primary cultures. The authors show that neural activity is also impacted in the bRAF-CA experiments with lower action potential spiking frequencies. In addition, MGE cultures treated with KCL to increase neural activity show induction of SST expression. However, treatment of Selumetinib and KCL did not result in induction of SST expression suggesting a critical role in the MAPK pathway.

This manuscript reveals new information in the field of cortical interneuron development and RAS/MAPK signaling. The findings show common phenotypes caused by increased RAS/MAPK signaling during cortical interneuron development and reveal mechanisms that link the impact of abnormal MAPK signaling with transcription factor expression in cINs.

The data are important for two key reasons:

1. -revealing new basic mechanisms key to understanding cortical interneuron development.

2. -describing novel phenotypes observed after RAS/MAPK pathway activation which is relevant to RASopathy syndromes that exhibit neurocognitive abnormalities.

The experiments are clearly described, and the data are of high quality. However, I have a few points the authors should address or consider in the manuscript. I also have a couple minor points for the authors to address.

Reviewer 2 Comments for the author...
Major Points:
1) -The authors show that Gad65/67 levels are increased in the E15.5 Nf1 loss or bRaf-ca- using western blot analysis. However, the dissection was the entire forebrain including cortex and striatum. Can the authors show or comment if the distribution of Gad65 or Gad67 cells are changed

© 2023. Published by The Company of Biologists under the terms of the Creative Commons Attribution License (https://creativecommons.org/licenses/by/4.0/).

in the cortex after Nf1 loss or bRaf-ca at embryonic stages. Is the increase in expression from increased positive cells in the ventral or dorsal telencephalon?

Response: This is a very good point, thank you! We tested a few GAD antibodies on E15.5 coronal brain sections and found a GAD67 antibody that worked well at this age. These data (new Supp. Fig. 4) demonstrate an upregulation of GAD67 signal in ventral domains but no change in dorsal regions containing migrating CINs. We have changed the text accordingly to reflect this in the results section. Since the increase in GAD67 does not seem to be impacting dorsal migrating CINs, this leads to a great transition to only focus on CIN relevant changes for this study.

2) -Related to the previous point, the authors show the reduction in Lhx6 expression but normal tdTomato expression at E15.5 in bRaf-ca embryos. The images in Supp. Fig4 (J compared to G) look to have increase tdTomato expression in the outer migratory pathway. It might be informative to examine the outer and inner migratory pathways of cINs. In fact, the outer cells appear to be the more sensitive region for the increased Satb1 cells in Figure 6. Can the authors comment if the Satb1 (and dtTomato fate map) have regional difference in the migratory pathway going to the cortex?

Response: This is interesting, thank you for noticing this. We went back to our images and counted upper layer vs. lower migrating cells using our E15.5 SATB1 labeling data (edited figure 6) for both tdTomato and SATB1+ cells. We found no changes in absolute tdTomato+ cell numbers between dorsal and ventral streams (we will report as data not shown to save room in the figure). However, this point is well taken, as the distribution of SATB1 expressing cells was highly enriched in the dorsal stream but less in ventral. Notably, the Nf1 mutants have slightly elevated numbers in the ventral stream, suggesting some divergence of the genotypes.

3) -The authors should consider some discussion about the models used in this study. For example, the bRAF activation model relies on the V600E mutation observed in cancers and the NF1 results are largely presented from biallelic deletion and not heterozygous. Do the authors think there is a necessary threshold of MAPK pathway activation for distinct phenotypes? What about other models of RAS/MAPK activation that model RASopathy syndromes. Potentially this point could be expanded in the discussion.

Response: We agree and have added more text in the discussion about the models.

4) -The authors make the following point in the last paragraph of the discussion: “This was not observed in the neocortex of bRaf hyperactive mutants (Data not shown) and could indicate other Nf1-regulated events 7 may influence ventral-derived oligodendrocytes”However, the authors do not show any data on the oligodendrocyte analysis including what markers were used and what stages were analyzed. If the authors want to include this statement, then this data would need to be included in the results section or supp Data section. Alternatively, the authors could expand the discussion to compare recent RASopathy/MAPK activation models and the effect on oligodendrocytes vs cINs (or other cells).

Response: Great point. For this paper, we have modified this text and have emphasized the common changes between the models, as this was the goal herein. We do agree however, this needs to be addressed and have a follow up paper looking into the various impacts on glia in a multitude of RAS/MAPK models. Our rewording acknowledges our previous published work showing Nf1 cKOs have elevated oligos in the transplants but does not delve into oligo changes in the bRafca model for simplicity.

5) -the authors use both Nkx2.1-cre (Fig. 1) and Lhx6-cre (supp fig. 2) to parse progenitor vs postmitotic targeting of NF1 loss or Braf-ca in the manuscript. The authors should clearly describe which cre is used for Figure 3. One option is to include a sentence after the Lhx6-cre description for the supp. data that all remaining figures are on the Nkx2.1-cre driven deletion/activation. Also, do the Lhx6-cre Nf1 cKOs survive to E18? Can the authors comment in more detail on how the phenotype is different from the Nkx2.1-cre experiments? This is an important point given the difference in timing for cre expression (Nkx2.1-cre early and Lhx6-cre in post-mitotic cINs).

© 2023. Published by The Company of Biologists under the terms of the Creative Commons Attribution License (https://creativecommons.org/licenses/by/4.0/).

Response: Thank you, the progenitor vs. post-mitotic question is a major point we have worked towards for the revision. During the pandemic, we had to drastically cut our mouse lines and lost a lot of money. This resulted in a loss of many mouse lines, including the Lhx6-Cre line, which was already difficult to acquire from fellow external labs (not commercially available). Unfortunately, we are not able to answer questions using this Cre line at this time.

However, to address the progenitor vs. post-mitotic question, we instead used a lentiviral strategy to deliver Cre to E13.5 MGE cells and perform similar transplant studies as in Figure 1. Lentiviruses take 2-3 days post transduction before their genetic cargo begins to express and in addition, we included the GABAergic biased Dlxi1/2b enhancer to drive Cre, which will delay when the cells can express Cre, beginning in post-mitotic GABAergic neurons. We can recapitulate the Nkx2.1-Cre SST and PV phenotypes shown in Figure 1 in the lentiviral-transduced transplanted cells with this strategy, suggesting these phenotypes are not restricted only to MGE progenitors. These data are now new Figure 2.

To clarify which Cre was used for each figure, we added your suggestion to the text to indicate that after Supp. Fig 2, all remaining figures utilize the Nkx2.1-Cre. Nkx2.1-Cre text was also added to the schema in Fig. 3.

Minor Points:

6) -could the authors comment if the morphology is changed in the in vitro SST+ cells in Braf-ca compared to NF1 loss or control in Figure 3B-D.

Response: Yes, although the in vitro data presented is not representative of cell size due to the massive increase of SST expression in the bRaf mutants. Instead, we measured the terminal point in our analyses, transplanted cells at maturity. We measured soma size and found that that the bRaf mutant cells were significantly larger and the Nf1 mutants were similar to WTs. This is new supplemental figure 2.

7) -Have the authors examined MAPK pathway activation in the MGE cultures or transplant experiments. Do the increased SST+ cells express phosphor-ERK1/2 or MAPK target genes?

Response: Unfortunately, we have had great difficulty doing this but agree it would be great to know. Basically, we need antigen retrieval to see reliable pERK signal but this kills the tdTomato signal and disrupts the SST signal. In contrast, we can’t see any pERK signal when no antigen retrieval is done. We have also tried other pMAPK pathway antibodies without success and don’t feel comfortable publishing any of these data.

8) -there are several Supp. Data figures. The authors could consider adding the cell death data (Supp figure 3) or ARX data (Supp figure 5) to the main manuscript.

Response: Thank you for this recommendation. We have now incorporated these data into the main document. The cell death data are now part of Figure 3, panels S and T; images were removed to facilitate making the panels of the figure fit. The ARX data are now new main Figure 7.

Reviewer 3 Advance summary and potential significance to field...
This manuscript examines the role of MapK signaling on the maturation of GABAergic cortical interneurons (CINs). The authors utilize two genetic models that result in increased RAS/MapK signaling including Nf1 loss of function (LOF) and bRaf constitutively active (bRaf-ca) mutant mice to examine postnatal maturation of CINs. Since these mice either don’t survive well postnatally (bRaf-ca) or show defects also in non-CINs (e.g. oligos in Nf1 LOF), they make use of a clever transplantation paradigm implanting embryonic MGE progenitors into the postnatal cortex and allow the mutant CINs mature. MGE cells from both genetic models show a reduction in the parvalbumin CINs and a concomitant increase in those expressing Somatostatin (SST) which also exhibit SST-like physiological characteristics, at least at early postnatal time points. Interestingly, they found that bRaf-ca mutant CINs elude cell death resulting in the maintained increases in SST+ CINs in the more mature brains. Western blots of embryonic forebrain show increased Gad65/67 levels indicating enhanced GABAergic differentiation. While Dlx proteins are known to specify the

© 2023. Published by The Company of Biologists under the terms of the Creative Commons Attribution License (https://creativecommons.org/licenses/by/4.0/).

GABAergic fate of CINs by directly binding the Gad genes, the authors observed no change in their expression, supporting an independent role for RAS/MapK signaling in GABAergic maturation of CINs. Finally, the authors provide data that support neuronal activity-driven MapK signaling leading to the increased GABAergic maturation and also SST expression (via Satb1). Taken together, these findings have relevance for the cognitive deficits observed in patients suffering from RASopathies.

Reviewer 3 Comments for the author...
This is a well-done study that provides important information for the RAS/MapK and forebrain development fields and thus appears appropriate for publication in Development, however, there are few issues that the authors need to address first.

1) While the authors cite their previous papers using a similar transplantation approach, this manuscript would benefit from more details being provided as to the experiments both in the results and methods sections. For example, I assume that MGEs from the same embryo are dissociated and transplanted into separate animals (postnatal day?) with the genotyping being carried out after the fact.

Response: Yes, thank you. Other reviewers also noted this, this was an oversight and we apologize. Detailed methods have now been added to the manuscript.

2) The question of, at what point in the MGE CIN lineage these changes in RAS/MapK signaling impact CIN subtype maturation, is interesting but not well addressed. Why wasn't the transplantation technique used for Lhx6-cre experiments, given the observed lethality? Is the Nf1 het an appropriate way to asses this? Ultimately, it would be more informative to be able to assess PV/SST proportions in mutant CINs after Lhx6-cre (i.e. postmitotic) recombination. As it stands, the question of whether Nf1 LOF or bRaf-ca in dividing MGE progenitors vs postmitotic CINs contributes to the observed changes in mutant CINs, remains unresolved.

Response: Yes, this is very important and we have worked to answer this. Please see our response #5 to reviewer 2 on the same matter.

3) The Satb1 data in Figure 5 would benefit from a more nuanced analysis. While it looks like a similar density of Satb1+ cells occupy the most superficial portion of the cortical plate in both mutants, the intermediate zone shows differential numbers – with more in the bRaf-ca mutants. If this difference is real, it may hint towards the distinct phenotypes seen in the two genetic models.

Response: Yes, we performed further analyses and this is correct. Both mutants exhibit an elevated SATB1 expression population in dorsal streams but only the Nf1 mutants have elevated numbers in the ventral stream. While we don’t understand what this means yet it is important to report for future researchers.

4) In my opinion, the Arx data should be included in the main figures as they show a strong and shared phenotype in mutant CINs between the 2 models.

Response: Yes, we agree and was also suggested by reviewer 2. This is now main Figure 7.

5) The SST data in vitro are pretty convincing, however, no assessment of PV+ CINs is performed. Can you detect PV+ CINs in vitro and if so, is their phenotype (or ratios) impacted when MapK signaling/neuronal activity is altered?

Response: We would love to do this, however, due to PV expression beginning so late during development (P12-14) in mouse cortical interneurons (3+ equivalent weeks in vitro), in vitro experiments become incredibly hard to interpret and do for this measurement. We also tried to examine MEF2C protein during these early stages, as it seems to be an early marker of PV-fated CINs, however, we could not detect the protein co-labeled with MGE-lineages in early postnatal CINs of the neocortex, despite RNA being reported in these cells. Thus, we don’t feel confident in providing a good readout to answer the question at this time.

© 2023. Published by The Company of Biologists under the terms of the Creative Commons Attribution License (https://creativecommons.org/licenses/by/4.0/).

Second decision letter

MS ID#: DEVELOP/2022/201371

MS TITLE: Distinct hyperactive RAS/MAPK alleles converge on common GABAergic interneuron core programs

AUTHORS: Sara J Knowles, April M Stafford, Tariq Zaman, Kartik Angara, Michael R Williams, Jason M Newbern, and Daniel Vogt

I have now received all the referees reports on the above manuscript, and have reached a decision. The referees' comments are appended below, or you can access them online: please go to BenchPress and click on the 'Manuscripts with Decisions' queue in the Author Area.

The overall evaluation is positive and we would like to publish a revised manuscript in Development, provided that the referees' comments can be satisfactorily addressed. Please attend to all of the reviewers' comments in your revised manuscript and detail them in your point-by-point response. If you do not agree with any of their criticisms or suggestions explain clearly why this is so. If it would be helpful, you are welcome to contact us to discuss your revision in greater detail. Please send us a point-by-point response indicating your plans for addressing the referee’s comments, and we will look over this and provide further guidance.

Reviewer 2

Advance summary and potential significance to field

Summary from first review has not changed. Significance copied below from first review:

This manuscript reveals new mechanistic information in the field of cortical interneuron development and RAS/MAPK signaling. The findings show common phenotypes caused by increased RAS/MAPK signaling during cortical interneuron development and reveal mechanisms that link the impact of abnormal MAPK signaling with transcription factor expression in cINs.

The data are important for two key reasons:

1. -revealing new basic mechanisms key to understanding cortical interneuron development.

2. -describing novel phenotypes observed after RAS/MAPK pathway activation which is relevant to RASopathy syndromes that exhibit neurocognitive abnormalities.

Comments for the author

The authors addressed all of my major and minor points from the first review. I do not have additional questions or comments. The revised manuscript is improved and shows robust data. Conclusions from this manuscript are relevant to better understand molecular mechanisms driving the development of cortical interneurons and provide new information on the overall impact of hyperactive RAS/MAPK signaling in the brain.

Reviewer 3

Advance summary and potential significance to field

The authors have done a good job revising the manuscript in accordance with the reviewers' comments. I feel that the revised paper represents an important contribution to the field and will be highly useful to the readers of Development.
Thus I support publication with a minor revision requested below.

© 2023. Published by The Company of Biologists under the terms of the Creative Commons Attribution License (https://creativecommons.org/licenses/by/4.0/).

Comments for the author

One of my concerns in the previous version of the manuscript was how the question of RAS/MapK in progenitors vs post-mitotic neurons was addressed. The authors mentioned that they no longer have the Lhx6-cre mice and are thus unable to carry out the requested transplantation studies. I appreciate that they have taken a different approach and used a lentiviral approach with the Dlx1/2 enhancer to drive cre specifically in the GABAergic neurons. They state that this will only drive recombination in post-mtotic MGE cells but I didn't see any data in the manuscript to support that or in their 2015 Cell Reports paper. I tend to agree with their logic that by the time cre is expressed, using the lentiviral approach the MGE cells will have left the cell cycle but without supporting data of this they should at least discuss this potential caveat. I don't need to see a revised manuscript if the editor is fine with confirming that such a brief comment is added to the Discussion.

Second revision

Author response to reviewers' comments

We agree that some text should be added to the discussion to address a caveat. This has been done.

Third decision letter

MS ID#: DEVELOP/2022/201371

MS TITLE: Distinct hyperactive RAS/MAPK alleles converge on common GABAergic interneuron core programs

AUTHORS: Sara J Knowles, April M Stafford, Tariq Zaman, Kartik Angara, Michael R Williams, Jason M Newbern, and Daniel Vogt
ARTICLE TYPE: Research Article

I am happy to tell you that your manuscript has been accepted for publication in Development, pending our standard ethics checks.

© 2023. Published by The Company of Biologists under the terms of the Creative Commons Attribution License (https://creativecommons.org/licenses/by/4.0/).
